# The role of pubertal development in the association between trauma and internalising symptoms in female youth

**DOI:** 10.1111/jcpp.14139

**Published:** 2025-03-04

**Authors:** Niamh MacSweeney, Phoebe Thomson, Tilmann von Soest, Christian K. Tamnes, Divyangana Rakesh

**Affiliations:** ^1^ Division of Mental Health and Substance Abuse Diakonhjemmet Hospital Oslo Norway; ^2^ Department of Psychology, PROMENTA Research Centre University of Oslo Oslo Norway; ^3^ Norwegian Centre for Mental Disorders Research, Institute of Clinical Medicine University of Oslo Oslo Norway; ^4^ Autism Center, Child Mind Institute New York NY USA; ^5^ Neuroimaging Department, Institute of Psychology, Psychiatry & Neuroscience King's College London London UK

**Keywords:** Trauma, puberty, internalising symptoms, pubertal timing, pubertal tempo, longitudinal

## Abstract

**Background:**

Exposure to trauma in childhood is associated with an increased risk for internalising symptoms. Alterations in pubertal development has been proposed as a potential mechanism underpinning this association. However, longitudinal studies, which are needed to examine pubertal development over time, are scarce. The goal of this pre‐registered study was to examine how trauma exposure shapes the timing and tempo of pubertal development, and in turn contributes to risk for internalising symptoms in female youth.

**Methods:**

Using the largest longitudinal sample to date, we characterised profiles of pubertal development across four time points in female youth from the Adolescent Brain Cognitive Development (ABCD) Study (*N* = 4,225, age range = 9–14 years) using latent profile analysis. Pubertal development was assessed using the Pubertal Development Scale (at four time points). Trauma exposure was quantified using the post‐traumatic stress disorder subscale from the parent‐report Kiddie Schedule for Affective Disorders and Schizophrenia for DSM‐5 (at baseline), and internalising symptoms were assessed using the self‐report Brief Problem Monitor (at 3‐year follow‐up).

**Results:**

Pubertal development could be grouped into three latent classes: early starters (9% of sample), typical developers (76%) and slow developers (15%). The early starters demonstrated higher levels of trauma exposure compared to typical developers and slow developers, while slow developers showed the least exposure to trauma. Youth with greater exposure to trauma were at an increased risk for internalising symptoms at ages 12–14 years, and this association was mediated by a higher pubertal status at ages 9–10 years, but not by a faster pubertal tempo.

**Conclusions:**

Accelerated pubertal development, characterised by an earlier age of onset but not a higher pubertal tempo in the transition from late childhood to early adolescence, may be a mechanism through which trauma exposure in childhood increases risk for internalising symptoms in female youth.

## Introduction

Exposure to early life trauma is associated with substantially increased risk for later depression and anxiety (Kessler et al., 2010). However, the biological mechanisms of this association remain unclear. It is suggested that early life trauma initiates a sequence of physiological changes during adolescence that may contribute to the onset of internalising symptoms. One proposed mechanism in this pathway involves alterations in pubertal development during adolescence as a consequence of prior trauma exposure (Colich & McLaughlin, 2022).

Puberty is widely recognised to be the catalyst for the substantial biological, psychological, and social changes that characterise adolescence (Pfeifer & Allen, 2021). Although many youths manage to navigate the changes that occur during this period, unfortunately, a significant proportion do not. Indeed, adolescence is a life stage marked by the emergence of depression and some anxiety‐related disorders (Solmi et al., 2022). This is particularly notable for females who are twice as likely to experience internalising symptoms, including depression and anxiety, compared to males. This sex‐difference first arises around puberty (Platt, Bates, Jager, McLaughlin, & Keyes, 2021) with recent evidence suggesting that the incidence of internalising symptoms in female adolescents is increasing (Keyes & Platt, 2023). Further, cross‐sectional studies have shown that adolescents, particularly females, who begin puberty ahead of their same‐age, same‐sex peers are at an increased risk for internalising symptoms (MacSweeney et al., 2023; McNeilly, Saragosa‐Harris, Mills, Dahl, & Magis‐Weinberg, 2022; Ullsperger & Nikolas, 2017).

A host of interacting biological factors such as genetics and neurobiology, and socio‐environmental factors including early life stress, are thought to contribute to the early onset of puberty, which may in turn increase risk for internalising symptoms (Pfeifer & Allen, 2021; Thapar, Eyre, Patel, & Brent, 2022). Among these factors, threat exposure has been suggested to play a particularly central role. A recent meta‐analysis showed that threat exposure is an important predictor of the early onset of puberty (Colich, Rosen, Williams, & McLaughlin, 2020), which may, in turn, contribute to the onset of internalising symptoms during adolescence. These findings, while largely based on cross‐sectional evidence that does not examine pubertal development over time, are consistent with life history theory, which posits that the length of childhood and pubertal onset could be determined (to some extent) by environmental experiences (Belsky, Steinberg, & Draper, 1991; Ellis, Figueredo, Brumbach, & Schlomer, 2009). In this framework, exposure to threat is purported to accelerate the timing and pace of pubertal maturation to maximise chances of reproduction prior to potential mortality. Although life history theory also posits that adversity characterised by deprivation (e.g. emotional and physical neglect) is related to delayed pubertal maturation (Ellis, 2013; Ellis et al., 2009), recent meta‐analytic work did not find support for this hypothesis (Colich et al., 2020). However, more longitudinal research is needed to better understand how exposure to trauma, and its subtypes, may shape pubertal *development* over time, and the associated risk for the development of internalising symptoms.

Importantly, pubertal maturation varies substantially in timing and tempo across individuals (Dorn & Biro, 2011). Although some longitudinal work suggests that earlier timing and faster tempo are associated with increased internalising problems in females (Marceau, Ram, Houts, Grimm, & Susman, 2011), the extant literature is largely cross‐sectional. It remains therefore unclear whether earlier onset of puberty (i.e. pubertal timing), faster progression (i.e. pubertal tempo), or a combination of both are most relevant for mental health. Additionally, how prior trauma exposure relates to pubertal development and the female‐specific vulnerability to internalising symptoms in adolescence (McGrath et al., 2023) remains poorly understood. The current study addresses these knowledge gaps by investigating (a) whether trauma exposure is associated with specific profiles of pubertal development (e.g. earlier pubertal timing *and/or* faster pubertal tempo compared to normative pubertal development) and (b) whether specific pubertal development profiles characterise female youth who are at risk for developing internalising symptoms. This work may help identify youth who may be most in need of additional support during adolescence.

We leveraged recently available longitudinal data across early adolescence (when most females are undergoing pubertal transitions) from the large Adolescent Brain Cognitive Development (ABCD) Study. Specifically, we examined how exposure to trauma in childhood relates to different patterns of pubertal development, including pubertal status at ages 9–10 years and the rate at which females progress through pubertal maturation between the ages 9–14. We then tested how these patterns of pubertal development relate to later internalising symptoms. Given the heightened risk of internalising disorders observed specifically among female youth (Keyes & Platt, 2023), our primary analyses focused on our pre‐registered aims within a female‐only sample. We conducted additional exploratory analyses in a male sample for which we did not have pre‐registered hypotheses. In females, we hypothesised that greater exposure to trauma in childhood would be associated with a more accelerated pattern of pubertal development, characterised by a more advanced pubertal status as well as a faster pace of development over time. We also hypothesised that this pattern of pubertal development would mediate the positive association between childhood trauma exposure and later internalising symptoms in females.

## Materials and methods

The data analysis plans for this study were preregistered on the Open Science Framework (https://osf.io/9azuj). Any deviations from our preregistered plan have been fully described.

### Sample

This study used data (release 5.0) from the Adolescent Brain Cognitive Development (ABCD) Study®. The ABCD Study recruited ~11,800 9‐ to 10‐year‐olds at baseline between 2016 and 2018 from 21 sites across the United States (Casey et al., 2018; Garavan et al., 2018). The baseline cohort is being followed up for ten years with puberty data collected annually, as well as mental health assessments every 6 months. Here, we used data collected from youth at baseline (T1; *M*_Age_ = 9.88 years), year 1 (T2; *M*_Age_ = 10.88 years), year 2 (T3; *M*_Age_ = 11.98 years), and year 3 (T4; *M*_Age_ = 12.87 years) assessments. The sample used in the current analyses comprised *N* = 4,225 unrelated female youth (based on parent‐reported biological sex at birth). One child was randomly selected from each family. All variable names used can be found in Table S1.

### Measures

#### Trauma exposure

Exposure to traumatic events was assessed using the parent‐report post‐traumatic stress disorder (PTSD) subscale from the Kiddie Schedule for Affective Disorders and Schizophrenia for DSM‐5 (KSADS; Kaufman et al., 1997); administered at T1. See Thompson et al. (2022) and Supporting Information (SI): Section 1 for details of the type of trauma data available in ABCD. We generated a sum score of the number of traumatic events based on 17 different experiences rated as either 0 (has not experienced) or 1 (has experienced). Given the skewed nature of the trauma variable in the ABCD sample (see Figure S1), all values above two were recoded as two, and trauma exposure was modelled as a continuous variable. This is a slight deviation from our preregistration where we stated that trauma would be operationalised as a categorical variable containing three levels: no exposure (0 events), mild exposure (1 event) and moderate exposure (≥2 events). This change was made due to the complications associated with using nominal predictors in mediation models.

#### Pubertal maturation

The Pubertal Development Scale (PDS; Petersen, Crockett, Richards, & Boxer, 1988) was used to examine the perceived development of secondary sex characteristics such as growth spurts, body hair growth, skin changes, breast development and menarche in females and voice changes and facial hair growth in males. The PDS includes five items, and each characteristic is rated on a 4‐point scale (1 = no development; 2 = development has barely begun; 3 = development is definitely underway; and 4 = development is complete; except menstruation, which is coded 1 = has not begun, 4 = has begun). We used average PDS scores from T1, T2, T3 and T4 as our measure of perceived physical pubertal development at each time point (see Supporting Information for how missingness was handled). Higher scores reflect more advanced pubertal maturation. Consistent with other studies (Cheng et al., 2021; Holm et al., 2023; MacSweeney et al., 2023; McNeilly et al., 2022), given the significant number of ‘I don't know’ responses for youth‐report PDS in the early waves of ABCD data collection, caregiver‐report PDS was used instead of youth self‐report. Notably, both parent‐report and youth self‐report PDS have been found to align with clinician rated Tanner Staging (Koopman‐Verhoeff, Gredvig‐Ardito, Barker, Saletin, & Carskadon, 2020). See the data analysis section for how scores of pubertal status and tempo were derived using PDS data.

#### Internalising symptoms

Youth internalising symptoms were assessed using the sum score for the internalising subscale from the youth‐report Brief Problem Monitor (BPM; Achenbach, 2009), at T4 (youth aged 12–14 years, which is the last time point with complete data currently available in ABCD). The BPM examines youth behaviour and symptoms over the past week and comprises 9 items rated as 0 (‘not true’), 1 (‘somewhat true’) or 2 (‘very true’). Due to the skewed distribution of the BPM data (see Figure S4), BPM scores were transformed using the Yeo Johnson method (*bestNormalise* package in R).

### Data analysis

#### Deriving patterns of pubertal maturation using latent profile analysis

Latent profiles (using average PDS scores for each participant at each time point as input values) were computed using the *lcmm* package in R (version 4.3.0) to model patterns of pubertal maturation. First, to determine the optimal number of classes (i.e. profiles characterising pubertal development), we estimated models with no within‐class variance, using class numbers ranging from one to six. The best fitting models were chosen based on recommended criteria, including the lowest Bayesian information criterion (BIC) and Akaike information criterion (AIC) values, entropy values closest to 1, and a sufficient percentage (≥5%) of individuals within each class (Jung & Wickrama, 2008). To obtain the final model, we estimated the two best fitting class solutions with a random intercept and slope to examine within‐class variance (random starts = 100 and maximum iterations = 10). Age was centred on the average age at T1 (9.88 years). Not all participants had complete follow‐up puberty data at each of the four time points. As per the requirements of the *lcmm* package, observations with missing puberty data (1399/16856 observations) were omitted from the latent profile analysis.

To test whether latent classes characterised by more accelerated pubertal development had higher trauma exposure, we used Kruskal–Wallis tests with Dunn's tests for post hoc pair‐wise comparisons due to the non‐normality and non‐homogeneity of variance in our data. We used the same procedure to examine whether the latent classes associated with accelerated pubertal development exhibited elevated levels of internalising symptoms. This allowed us to gain insight into whether individuals with different profiles of pubertal status and tempo also demonstrate differences in trauma exposure and internalising symptoms. We then used structural equation modelling (SEM) (via the *lavaan* package in R) to investigate whether individual‐level intercept and individual‐level slope values, derived from our latent profile analysis (i.e. used as manifest variables) and reflecting pubertal status at baseline and pubertal tempo between ages 9 and 14, mediated the relationship between childhood trauma exposure and internalising symptoms among participants aged 12–14 years. The mediation model was run with a fixed effect of age, bootstrapping (5,000 draws), which accounts for the non‐normality of the data, and bias‐corrected standard errors. Beta estimates reported are standardised. Statistical significance was evaluated using a *p* value of ≤.05 and bias‐corrected confidence intervals.

#### Sensitivity analyses

Body mass index (BMI), household income and race/ethnicity were included as covariates in sensitivity analyses due to the association between these factors and risk for internalising symptoms (Peverill et al., 2021; Quek, Tam, Zhang, & Ho, 2017). Although not part of our preregistered analyses, we re‐ran our multiple mediation model including earlier youth internalising symptoms (available at the 6‐month follow‐up from baseline) as a covariate. This allowed us to examine whether trauma exposure was associated with the *emergence* of internalising symptoms in early adolescence and the role of pubertal development in this association. To examine whether using a recoded trauma variable (range 0–2) affected our results, we also re‐estimated our models using the original KSADS PTSD sum score (range 0–17) as the predictor variable. Study site was not included as a covariate due to having interclass correlation coefficient (ICC) values close to zero (see Supporting Information: Section 4).

#### Exploratory analyses

To investigate the importance of the timing of pubertal timing measurement, we undertook mediation analyses to determine whether pubertal timing scores (derived from model residuals and modelled as a linear effect) at different ages mediated the association between early life trauma exposure and later internalising symptoms. In non‐pre‐registered exploratory analyses, we re‐ran our main analyses in male youth (*N* = 4,737) as per the methods described for the female‐only analyses. Our exploratory analyses are described in Supporting Information: Sections 5 and 6.

## Results

Characteristics of the study sample are presented in Table 1.

**Table 1:** Descriptive statistics for female sample

Characteristic	T1, *N* = 3,844^a^	T2, *N* = 3,756^a^	T3, *N* = 3,725^a^	T4, *N* = 3,668^a^
Age in years	9.88 (0.62)	10.88 (0.64)	11.98 (0.67)	12.87 (0.64)
Internalising symptoms (BPM)	—	—	—	2.61 (2.64)
Missing	—	—	—	198
Trauma exposure				
0 traumatic events	2,438/3,844 (63.4%)	—	—	—
1 traumatic event	1,033/3,844 (26.9%)	—	—	—
≥2 traumatic events	373/3,844 (9.7%)	—	—	—
PDS average score	1.75 (0.51)	2.07 (0.62)	2.54 (0.66)	2.93 (0.58)
Race/Ethnicity				
White	2,077/3,844 (54%)	—	—	—
Black	491/3,844 (12.8%)	—	—	—
Hispanic	757/3,844 (19.7%)	—	—	—
Asian	94/3,844 (2.4%)	—	—	—
Other	425/3,844 (11.1%)	—	—	—
Parental education				
Degree from 4‐year college or more	2,240/3,840 (58.3%)	—	—	—
Some college education	1,047/3,840 (27.3%)	—	—	—
HS graduate no college	357/3,840 (9.3%)	—	—	—
Without HS diploma	196/3,840 (5.1%)	—	—	—
Missing	4	—	—	—
Household income				
<$5,000	96/3,584 (2.7%)	—	—	—
$5,000–$11,999	116/3,584 (3.2%)	—	—	—
$12,000–$15,999	80/3,584 (2.2%)	—	—	—
$16,000–$24,999	158/3,584 (4.4%)	—	—	—
$25,000–$34,999	213/3,584 (5.9%)	—	—	—
$35,000–$49,999	292/3,584 (8.1%)	—	—	—
$50,000–$74,999	476/3,584 (13.3%)	—	—	—
$75,000–$99,999	551/3,584 (15.4%)	—	—	—
$100,000–$199,999	1,162/3,584 (32.4%)	—	—	—
>$200,000	440/3,584 (12.3%)	—	—	—
Missing	260	—	—	—

BPM, brief problem monitor; HS, high school; PDS, Pubertal Development Scale.

^a^ Mean (*SD*); *n*/*N*.

### Latent classes of pubertal development

The summary fit statistics for the latent class models with no within‐class variance are reported in Table S2. Based on the fit statistics (e.g. AIC, BIC), the three‐ and four‐class solutions were found to be the optimal models. When these solutions were run with within‐class variance, a three‐class solution demonstrated the best model fit across criteria. The class percentages and mean values for the intercept and slope for the 3‐class solution are presented in Table 2. The full solutions for the three‐class and four‐class models are detailed in Supporting Information: Section 2.

**Table 2:** Mean intercept and slope values and class sample percentages for a three‐class solution from the latent profile analysis (LPA)

Class	Mean intercept (*SD*)	Mean slope (*SD*)	% of sample (*N*)
Early starters	2.54 (6.43)	0.23 (0.32)	8.97 (379)
Typical developers	1.67 (2.57)	0.46 (0.67)	76.40 (3228)
Slow developers	1.37 (2.17)	0.18 (0.97)	14.63 (618)

Age was centred at the mean age at T1 (9.88 years) in the LPA, and therefore the mean intercept and slope values reported here correspond to when youth are aged 9.88 years. *SD*, standard deviation; *N* participants = 4,225; *N* observations = 15,457.

**Figure 1:**
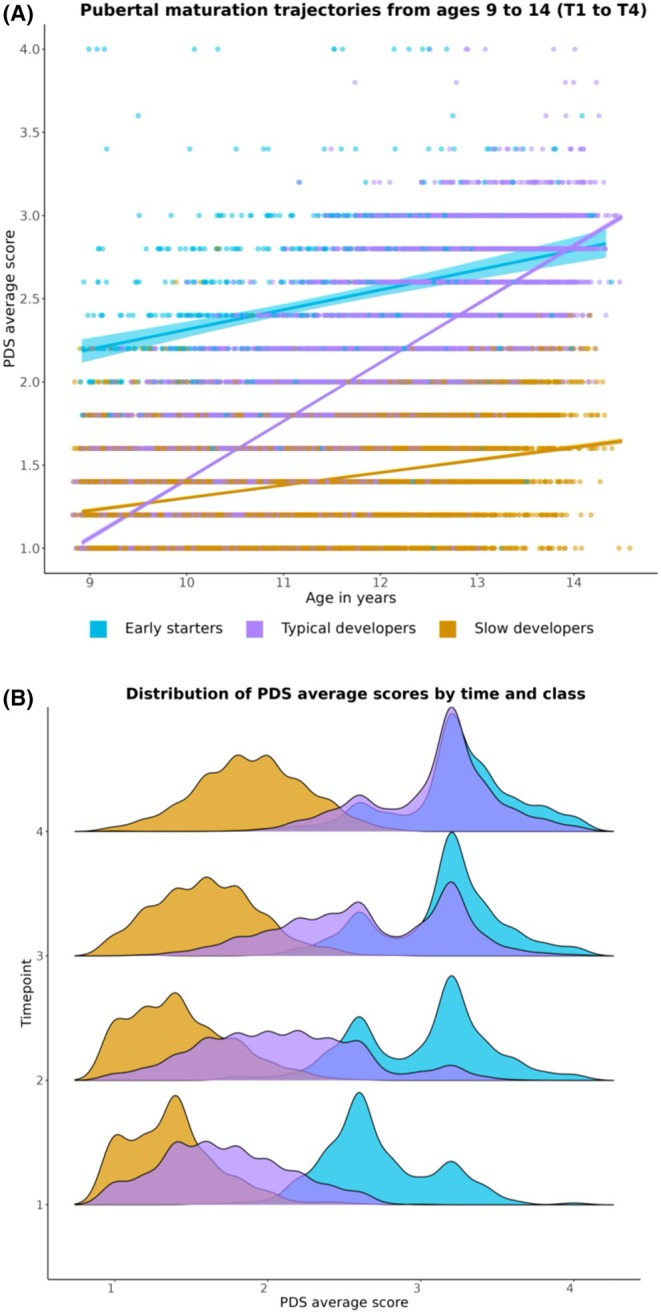
(A) Pubertal maturation trajectories from baseline (T1) to 3‐year follow‐up (T4), which spans ages 9–14 years. Regression lines represent the mean predicted linear growth trajectories for each puberty class with 95% confidence intervals, which are overlaid on individual raw data points (*N* = 4,225; observations = 15,457). Classes were derived using latent profile analysis with a random intercept and slope. For plotting purposes, we show the raw data used to derive the latent classes and plot the individual data points overlaid with a mean predicted linear regression for each class. (B) Ridge plots showing the variance of average pubertal development scores from T1 to T4 per puberty class. PDS, Pubertal Developmental Scale

Results demonstrate three distinct patterns of pubertal development (Figure 1): ‘typical developers’, ‘slow developers’ and ‘early starters’. Class labels were informed by prior work on puberty milestones (Marshall & Tanner, 1969; Mendle, Beltz, Carter, & Dorn, 2019; Tanner, 1986). Typical developers (76% of sample) were characterised by low intercept values (mean PDS = 1.67), indicating that they were in the early stages of puberty at T1 but had the most rapid pace of development over time (shown by the high mean slope value) such that they were in the later stages of puberty at T4. Slow developers (15% of sample) exhibited similar low intercept values at T1 (mean PDS = 1.37) but a lower slope value than typical developers, indicating a slower pace of pubertal maturation such that they were still in the early stages of puberty at T4. Finally, early starters (9% of sample) were characterised by more advanced pubertal maturation (mean PDS = 2.54) and were midway through puberty at T1 but showed a more protracted pace of development (i.e. lower slope value) over time such that their degree of pubertal maturation was like the typical developers at T4. Figure 1A,B illustrate the pubertal maturation trajectories and variance from T1 and T4 for each puberty class.

### Puberty class membership, trauma exposure, and internalising symptoms

**Figure 2:**
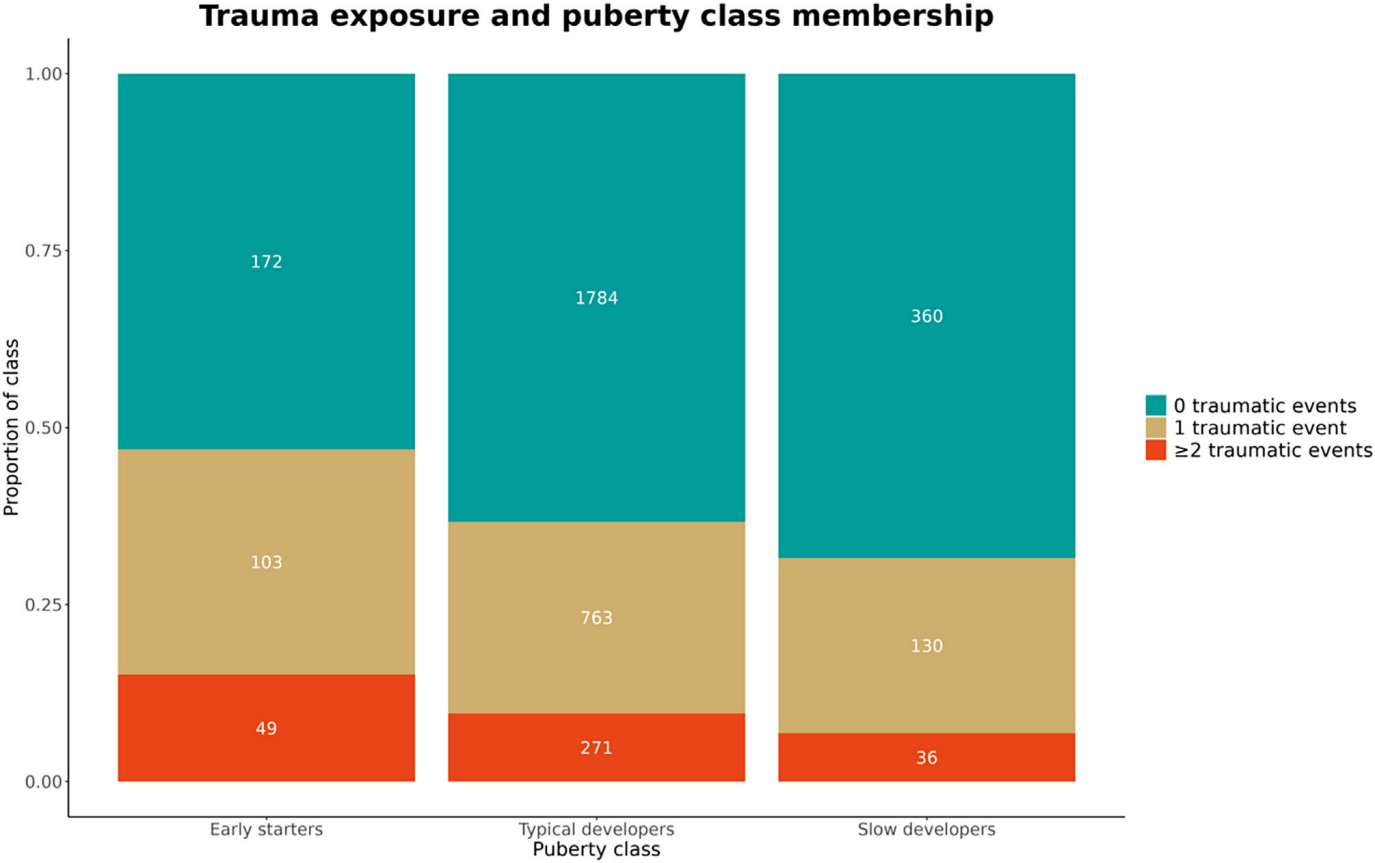
Proportion and frequency counts of participants with 0, 1 or ≥2 traumatic events reported in childhood, per puberty development class. *N* = 3,668

A statistically significant difference was found in the degree of trauma exposure between puberty development classes: *H*(2) = 23.83, *p* < .001 (Figure 2). Dunn's post hoc tests (with Bonferroni correction) showed that early starters had significantly higher trauma exposure compared to typical developers (*p* < .001) and slow developers (*p* < .001) (See Table 3). Moreover, slow developers were found to have significantly lower trauma exposure compared to typical developers (*p =* .020).

**Table 3:** Sample size, mean trauma exposure and internalising symptoms, and standard deviations per puberty development class. Note: Sample sizes vary due to missing internalising symptoms (BPM) data

Class	Trauma exposure			Internalising symptoms		
	*N*	Mean	*SD*	*N*	Mean	*SD*
Early starters	324	1.62	0.74	297	2.91	2.69
Typical developers	2,818	1.46	0.66	2,676	2.70	2.69
Slow developers	526	1.38	0.61	497	1.99	2.25

Puberty classes differed significantly in levels of internalising symptoms: *H*(2) = 36.45, *p* < .001. Dunn's post hoc tests (with Bonferroni correction) demonstrated that slow developers had significantly lower internalising symptoms compared to early starters (*p* < .001) and typical developers (*p* < .001) (See Table 3). Internalising symptoms were not significantly different between early starters and typical developers (*p* = .18).

### Does puberty status or tempo mediate the association between childhood trauma exposure and later internalising symptoms?

**Figure 3:**
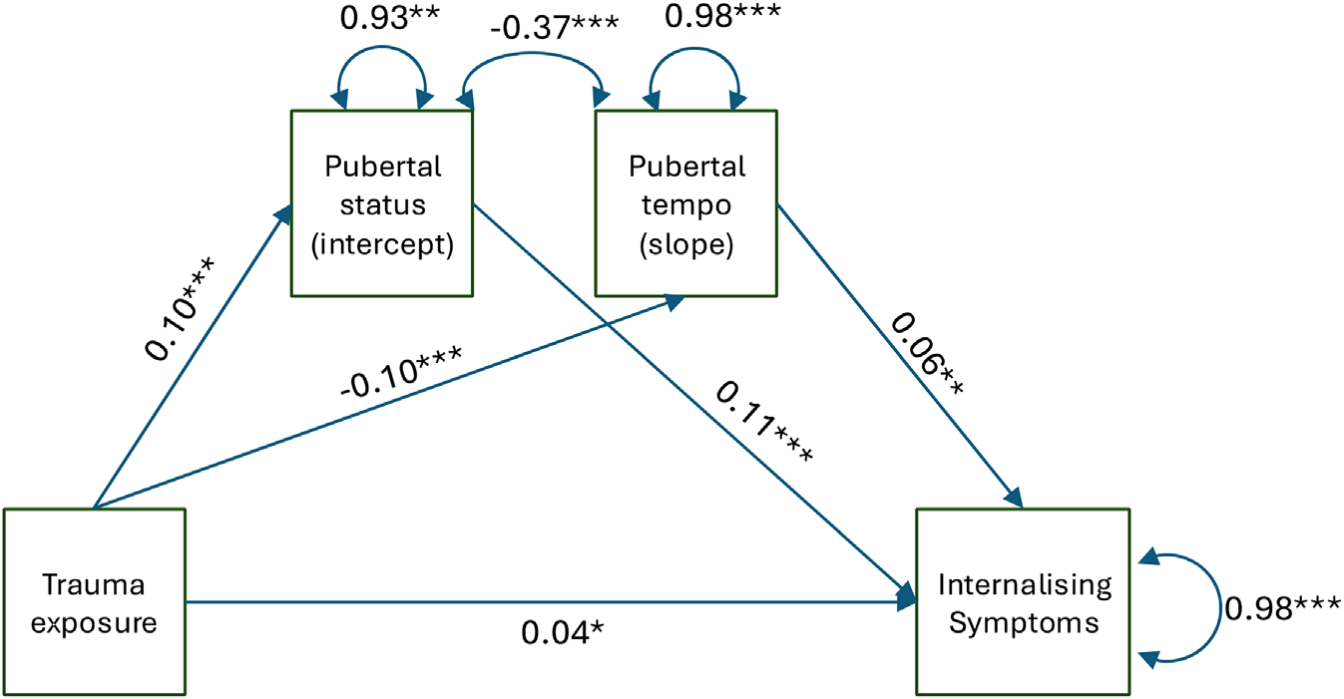
Multiple mediation model illustrating the relationships between trauma exposure (predictor), individual‐level intercept (pubertal status at T1), slope (pubertal tempo from T1 to T4) as mediators and internalising symptoms at T4 (outcome). Standardised regression coefficients are shown for each path. The residual variance of the mediator and outcome variables, indicated by the double‐headed curved arrows, reflects the variability not explained by the model. The double‐headed curved arrow between pubertal status and tempo reflects the correlation between the residuals of these two variables. Indirect standardised effects: Pubertal status (intercept): β = 0.01 (i.e. 0.10 × 0.11), Pubertal tempo: β = −0.006 (i.e. −0.10 × 0.06). * = significant at *p* ≤ .05, ** = significant at *p* ≤ .01, *** = significant at *p* ≤ .001. For clarity of plotting, the covariate of age was not included in the figure, but full details of the mediation model can be found in the Supporting Information

We used SEM to test whether pubertal status at ages 9–10 and tempo over ages 9–14, measured using individual‐level intercept and slope values, derived from the latent profile analysis, mediated the relationship between childhood trauma exposure and later internalising symptoms at T4. The model was just identified, having zero degrees of freedom, hence we do not report model fit statistics. We found a significant direct effect, suggesting that greater trauma exposure in childhood was associated with increased internalising symptoms (β = 0.037, *p* = .035, 95% CI [0.003, 0.105]). Further, a more advanced initial pubertal status (i.e. a higher intercept value of pubertal status) mediated the positive association between trauma exposure and internalising symptoms (indirect effect: β = 0.010, *p* < .001, 95% CI [0.009, 0.024]; Figure 3). We also found a significant indirect effect of pubertal tempo (β = −0.006, *p* = .005, 95% CI [−0.03, −0.006]; Figure 3). However, in this pathway, trauma exposure was linked to a slower tempo (i.e. a lower slope value of pubertal status), indicating that pubertal tempo acted as a suppressor in the association between trauma exposure and internalising symptoms in the multiple mediation model where both initial pubertal status and pubertal tempo were included simultaneously as mediators.

To examine whether a significant suppressor effect of pubertal tempo (i.e. slope) remained when not accounting for initial pubertal status (i.e. intercept), we ran separate mediation models where individual‐level intercept and slope values were tested as single mediators. In stand‐alone models, while initial pubertal status mediated the association between trauma exposure and internalising symptoms, as indicated by a significant indirect effect (β = 0.008, *p* = .003, 95% CI [0.006, 0.020]), pubertal tempo did not (β = −0.002, *p* = .308, 95% CI [−0.008, 0.002]). The intercept‐only and slope‐only mediation models are shown in Figures S7 and S8, respectively.

The full outputs for the mediation models are reported in Supporting Information: Section 3.

#### Sensitivity analyses

When we included participant BMI and household income as covariates in our multiple mediation model all effects remained significant. However, when race/ethnicity was included as an additional covariate, the direct effect was no longer significant, though the indirect effects remained significant. Study site was not included as a covariate due to having interclass correlation coefficient (ICC) values close to zero (see Supporting Information: Section 4). When earlier internalising symptoms (at the 6‐month follow‐up) was included in the main model, the significant indirect effects remained, although the direct effect between trauma exposure and later internalising symptoms was no longer significant. Analyses were re‐run using the original KSADS PTSD sum score as a continuous variable (range 0–17). Results were consistent with our main model. See Supporting Information: Section 4, for full details of the sensitivity analyses.

#### Exploratory analyses

The male results are detailed in Section 6, Supporting Information. In summary, we found that patterns of pubertal development in males could also be grouped into three classes: Early starters (5%), typical developers (51%) and slow developers (44%) (see Figure S12). Slow developers reported lower trauma exposure compared to the typical developers, but no other puberty class differences were found in terms of trauma exposure or internalising symptoms in males. In our mediation analyses, a significant direct effect was observed whereby greater trauma exposure was associated with higher levels of internalising symptoms (β = 0.080, *p* = .001, 95% CI [0.031, 0.129]). Pubertal status (individual‐level intercept values) but not pubertal tempo (individual‐level slope values) was found to mediate the association between trauma exposure and internalising symptoms in males (β = 0.005, *p* = .045, 95% CI [0.001, 0.010]).

After correction for multiple comparisons, we did not find any evidence for pubertal timing in any 6‐month age bin as a mediator between trauma exposure and later internalising symptoms, as indicated by the absence of a significant indirect effect (see Table S9).

## Discussion

In a large longitudinal sample, we examined how exposure to trauma in childhood relates to different patterns of pubertal development, and their associations with later internalising symptoms in female youth. We found that pubertal development could be grouped into three distinct classes: early starters, slow developers, and typical developers. The early starters (characterised by advanced initial pubertal status but relatively slower tempo) had higher levels of trauma exposure compared to both the typical developers and slow developers, while slow developers (characterised by low pubertal status initially and the slowest pubertal tempo over time) had the least exposure to trauma. The classes of youth with a more advanced initial pubertal status or higher pubertal tempo over time, namely the early starters and typical developers, respectively, had significantly higher mean levels of internalising symptoms compared to the slow developers. Mediation analyses indicated that females with greater exposure to trauma are at an increased risk for later internalising symptoms at ages 12–14 years, and that this association is partially explained by starting puberty ahead of their peers.

Using a data‐driven approach, we characterised individual differences in patterns of pubertal maturation in female youth between the ages 9–14 years in the largest longitudinal sample to date. Consistent with existing research on normative patterns of pubertal development, our results indicate that most female youth (76%) were in the pre‐to‐early stages of puberty initially (mean PDS = 1.67; development characterised as having not yet or barely begun) and were in the later stages of puberty at T4 (see Figure 1; development characterised as definitely underway or nearing completion) (Koopman‐Verhoeff et al., 2020; Marceau et al., 2011; Negriff, Blankson, & Trickett, 2015). A second group of females (15%) were also in the pre‐to‐early stages of puberty at baseline (mean PDS = 1.37) but demonstrated a slower pubertal tempo such that they were still in the early‐mid stages of puberty at T4. A smaller group of females (9%) were more advanced in their initial pubertal development (mean PDS = 2.54) but due to a slow tempo were at a similar level of pubertal maturation as typical developers at T4. Although longitudinal research on profiles of pubertal development is sparse, our findings align with prior work (Negriff et al., 2015) and could be used to inform future research to understand how patterns of pubertal maturation relate to other developmental outcomes.

Partially consistent with our hypothesis, and in line with prior meta‐analytic work and life history theory (Belsky et al., 1991; Colich et al., 2020; Ellis et al., 2009; Negriff et al., 2015; Senger‐Carpenter et al., 2024), we found that youth with higher exposure to trauma in childhood had a higher pubertal status at baseline, while youth with a slower pattern of pubertal development reported lower trauma exposure. Evidence, predominantly in rodents, suggests exposure to trauma in childhood may heighten the body's stress response and lead to the premature influx of adrenal and gonadal hormones that result in the early onset of puberty (Negriff et al., 2020). Recent ABCD studies have found support for accelerated neurodevelopment in response to stress (Callaghan & Tottenham, 2016), especially in fronto‐amygdalar networks (Thijssen, Collins, & Luciana, 2022; Vijayakumar, Whittle, & Silk, 2023). It has also been proposed that early puberty extends the period of increased sensitivity to sex‐steroid neural reorganisation that occurs during adolescence, which could underpin the increased risk for mental health difficulties observed in our study (Schulz, Molenda‐Figueira, & Sisk, 2009). Similarly, starting puberty later may be protective towards internalising symptoms due to a shortened window of sensitivity to sex‐steroid‐related neural changes. While longitudinal studies that directly test these biological mechanisms in humans are sorely needed, our study provides evidence for trauma‐associated alterations in female pubertal development.

Youth in the current study with higher childhood trauma exposure did not exhibit a more rapid pubertal tempo over time, which is in contrast to our hypotheses. Given that trauma was associated with advanced pubertal status at baseline, it is possible that trauma exposure contributed to accelerated pubertal maturation prior to the first measurement of puberty in the ABCD Study (i.e. before 9–10 years). Although there is little research on how early life adversity is associated with pubertal development earlier in childhood, one longitudinal study found that lower parental supportiveness and higher marital conflict during preschool ages was associated with more advanced pubertal maturation in females at age 7 (Ellis & Essex, 2007). Further, prior research suggests that when measured late in childhood, timing and tempo are likely to be inversely correlated (Keenan, Culbert, Grimm, Hipwell, & Stepp, 2014; Marceau et al., 2011). This is because youth who are more developed by late childhood have less pubertal development to complete in the same time as those just commencing puberty, leading to a lower rate of change (Negriff et al., 2015). Though future longitudinal studies capturing younger ages are needed to test whether adversity is associated with a faster pubertal tempo earlier in development, our findings suggest that higher childhood trauma exposure is associated with higher pubertal status in females by age 9.

Pubertal status at baseline, and pubertal tempo from ages 9 to 14, mediated the association between trauma exposure and later internalising symptoms. Importantly, in this multiple mediation model, trauma exposure was associated with a more advanced pubertal status at ages 9–10 but a slower pubertal tempo between ages 9 and 14 years, which in turn predicted higher internalising symptoms. Further, faster pubertal tempo was found to suppress the positive association between trauma exposure and internalising symptoms in this model. Although our findings are consistent with a recent systematic review on earlier pubertal development and internalising symptoms (Vijayakumar & Whittle, 2023), it is crucial to appreciate the individual differences that exist in these developmental pathways. For instance, the significant main effect of trauma exposure on internalising symptoms suggests that mechanisms other than or in addition to earlier pubertal timing are also at play. This raises the question of what differentiates the subset of trauma‐exposed adolescents who develop internalising symptoms *and* exhibit early pubertal development.

It has been proposed that the association between early pubertal timing and internalising symptoms is underpinned by an asynchrony between a young person's physical, cognitive and social development (Brooks‐Gunn, Petersen, & Eichorn, 1985; Ge & Natsuaki, 2009; Pfeifer & Allen, 2021). Importantly, the social environment plays an important role in mental health risk for early maturing youth (Vijayakumar & Whittle, 2023). For example, having a physical appearance that is different from one's peers could lead to increased risk for peer victimisation and result in higher levels of emotional distress for early‐maturing youth (Conley, Rudolph, & Bryant, 2012). While studies have examined neurodevelopmental alterations linking early adversity with psychopathology (Rakesh et al., 2021; Rakesh, Allen, & Whittle, 2023; Whittle, Zhang, & Rakesh, 2024), there is a paucity of longitudinal research that simultaneously examines how trauma and early puberty may contribute to neurodevelopmental differences that ultimately increase vulnerability to internalising symptoms. Additionally, the type of trauma experienced (e.g. threat vs. neglect) and the trajectory of internalising difficulties across adolescence (e.g. limited to early adolescence, persistent across adolescence, or only emerging in later adolescence) will be crucial to consider in future longitudinal research to better characterise at‐risk and resilient youth and inform prevention strategies (Ellis, Sheridan, Belsky, & McLaughlin, 2022; McLaughlin, Sheridan, Humphreys, Belsky, & Ellis, 2021; Vijayakumar & Whittle, 2023; Weavers et al., 2021). Moreover, this work could help refine existing theoretical frameworks, such as life history theory, so that they better reflect the nuances of how trauma exposure shapes the pace of biological ageing and mental health risk (Colich et al., 2020).

Contrary to our hypotheses, the association between trauma exposure and later internalising symptoms was not mediated by a faster pubertal tempo. These findings may reflect the more pubertally advanced youth having had a faster pubertal tempo earlier in development, but the age range of the ABCD sample did not allow us to test this hypothesis further. However, others have found that earlier pubertal timing *and* faster pubertal tempo were associated with increased risk for mood difficulties in a sample of black South African adolescents aged 10–15 (Kowalski et al., 2021). It is possible that the discrepancy in findings may be due to differences in the racial and ethnic composition between the two samples. The results from our sensitivity analyses suggest differences across racial and ethnic groups in how trauma exposure relates to pubertal maturation and risk for internalising difficulties, which could help explain the incongruent findings and highlights the importance of studying these associations in more diverse samples.

Youth in the slow developers class were less likely to experience internalising symptoms at ages 12–14 compared to the early starters and typical developers. This finding is consistent with prior work and suggests that late maturing females experience fewer internalising symptoms during adolescence because they have more time to assimilate to their surroundings, unlike their at‐risk early maturing peers (Brooks‐Gunn et al., 1985; Ge & Natsuaki, 2009). On the other hand, the typical developers and early starters did not differ significantly in their degree of internalising symptoms at ages 12–14. Although this finding was unexpected, the faster pace of pubertal development exhibited by the typical developers, compared to slow developers, could put them at an increased risk for internalising symptoms. Less widely investigated than pubertal timing, findings on pubertal tempo have been mixed, with studies reporting a positive association between pubertal tempo and depressive symptoms in females (Keenan et al., 2014; Marceau et al., 2011), as well as null effects (Mendle, Harden, Brooks‐Gunn, & Graber, 2010). However, work by Keenan and colleagues (Keenan et al., 2014) suggests that the developmental window during which symptoms are assessed is important for understanding how patterns of pubertal maturation relate to risk for internalising symptoms. For example, females with a faster pubertal tempo across the ages 9–17 were more likely to report an increase in depressive symptoms from ages 10 to 13, while a slower pubertal tempo was associated with a decrease in symptoms over time but higher depressive symptoms at age 10 (Keenan et al., 2014). These results may help explain the findings of the present study. Future work could build on our work by simultaneously examining patterns of pubertal development and depressive symptom trajectories over time. Together, these findings underscore the importance of studying the dynamic interplay of the biological and psychosocial factors that shape development across adolescence.

Although the large sample size and longitudinal design are strengths of the current study, there are limitations that must be considered. First, as the ABCD Study is a population‐based sample, the trauma data are heavily skewed with most youth reporting exposure to 0–2 traumatic events. Mere exposure to trauma does not necessarily mean that the event was perceived as traumatic and the types of traumatic events most reported in ABCD (e.g. death of a loved one; see Figure S2) may not align with clinical studies on trauma exposure. We also assessed trauma exposure at baseline, which may have led us to overlook or underestimate the effects of trauma that occurred at subsequent time points. Future research would benefit from assessing both different types of trauma exposure and pubertal development at multiple time points to estimate these associations longitudinally. Importantly, future research should also consider whether these events were *perceived* as traumatic and stressful. This will help provide a more comprehensive understanding of the longitudinal effects of trauma on pubertal maturation and internalising symptoms. Second, the peak age of onset for mood and anxiety‐related disorders tends to occur later in adolescence (>15 years), although some anxiety disorders can have an onset in mid‐childhood (Solmi et al., 2022). This suggests that there will likely be a greater incidence of internalising symptoms as the ABCD sample get older which can be studied in future ABCD data releases. Third, only linear associations were examined in the present study. As more time points of data become available in ABCD, future work should explore non‐linear methods of modelling pubertal development and also examine specific pubertal processes, such as gonadarche and adrenarche (Beltz, Corley, Bricker, Wadsworth, & Berenbaum, 2014; Marceau et al., 2011). Fourth, due to the high missingness in youth‐reported PDS, we used the parent‐report PDS to measure pubertal development, although parent and youth self‐report were found to be significantly correlated (Table S3). Subsequent studies should explore ways to combine parent and youth report to account for the role of self‐perceived pubertal development. Relatedly, Tanner Staging (Marshall & Tanner, 1969, 1970), the gold‐standard measure of pubertal development, is not available in the ABCD Study and results should be interpreted in light of the shortcomings of the PDS (see Mendle et al., 2019). Future researchers may benefit from converting the raw PDS scores available in ABCD into Tanner Stages (see Shirtcliff, Dahl, & Pollak, 2009) to provide more detailed information on pubertal status and to facilitate comparisons with studies that use Tanner Staging.

Finally, we focused on female youth in our main pre‐registered analyses. Albeit earlier in their pubertal development than females overall, males showed similar patterns of pubertal maturation. Greater trauma exposure was associated with increased internalising symptoms in males. Although a more advanced initial pubertal status was found to mediate this association, the statistical significance of this indirect effect was weak. Prior work suggests sex differences in how early‐life adversity may relate to stress reactivity, pubertal maturation (Fearon et al., 2017; Negriff et al., 2015) and risk for internalising problems (MacSweeney et al., 2023; McNeilly et al., 2022; Ullsperger & Nikolas, 2017). To test these sex differences, future longitudinal work should investigate these relationships when ABCD data spanning early to late adolescence are available.

Our results suggest that accelerated pubertal maturation characterised by an earlier age of onset but not a higher pubertal tempo across ages 9–14, may be a pathway through which exposure to trauma in childhood confers risk for internalising symptoms in female youth. Although the underlying biological mechanisms remain unclear, future studies that leverage longitudinal puberty and mental health measures can help advance our understanding of the factors that contribute to the development of internalising symptoms during adolescence, which could in turn have implications for preventative interventions for at‐risk youth.

## Code availability

All scripts used in the analyses for this project can be found on this GitHub repository: https://github.com/niamhmacsweeney/ABCDTraumaPubertyDepression.

**Key points**

- Childhood trauma is associated with risk for later internalising symptoms but the mechanisms underpinning this association remain largely unknown.
- Using longitudinal data, this study investigated the role of pubertal timing and tempo in the association between trauma exposure and later internalising difficulties in female youth.
- Structural equation modelling revealed an increased risk for internalising symptoms in trauma‐exposed youth in middle adolescence, which was partly explained by an earlier age of puberty‐onset but not by a faster pubertal tempo over time.
- These findings support the idea that accelerated pubertal maturation may be a pathway through which trauma exposure increases risk for internalising symptoms but further longitudinal studies are needed.
- Alterations in pubertal maturation are important to consider in the study of risk for internalising symptoms during adolescence, which have implications for preventative interventions for at‐risk youth.

## Supporting information


**Appendix S1.** Supporting information including sensitivity and exploratory analyses.

## Data Availability

To access data from the Adolescent Brain Cognitive Development^SM^ (ABCD) Study (https://abcdstudy.org), qualified researchers can apply for a data user certificate through their institution (https://nda.nih.gov/abcd/request‐access).
