# Supplementary material for: The role of pubertal development in the association between trauma and internalising symptoms in female youth
Source: J Child Psychol Psychiatry. 2025 Mar 4;66(8):1197–208. doi: 10.1111/jcpp.14139 (PMC12267676; doi:10.1111/jcpp.14139)
Supplement: Supplementary file 1 — Appendix S1. Supporting information including sensitivity and exploratory analyses. [file JCPP-66-1197-s001.docx]

Supplementary Information

The role of pubertal development in the association between trauma and internalising symptoms in female youth

MacSweeney et al.

Table of Contents

[Section 1: Data Cleaning 3](#_Toc178342204)

[ABCD variable names 3](#_Toc178342205)

[Trauma exposure data distribution 4](#_Toc178342206)

[Puberty data scoring and distribution 7](#_Toc178342207)

[Association between caregiver-report and youth-self report puberty measures 8](#_Toc178342208)

[Internalising difficulties data cleaning 9](#_Toc178342209)

[Section 2: Latent profile analysis results 10](#_Toc178342210)

[Latent profile analysis fit statistics with no within-class variance 10](#_Toc178342211)

[Latent profile analysis fit statistics with within-class variance 11](#_Toc178342212)

[Section 3: Mediation results 13](#_Toc178342213)

[Intercept stand-alone mediation model 15](#_Toc178342214)

[Slope stand-alone mediation model 17](#_Toc178342215)

[Section 4: Sensitivity analyses 19](#_Toc178342216)

[Additional covariates 19](#_Toc178342217)

[Section 5: Exploratory analyses 21](#_Toc178342218)

[The timing of pubertal timing 21](#_Toc178342219)

[Section 6: Male sample analysis 24](#_Toc178342220)

## Section 1: Data Cleaning

### ABCD variable names

| *Study characteristic* | *Variable name* | *Notes* |
| --- | --- | --- |
| Sex assigned at birth | demo_sex_v2 |  |
| Family ID | rel_family_id |  |
| Study site | site_id_l |  |
| Internalising symptoms | bpm_y_scr_internal_r |  |
| Trauma exposure | ksads_ptsd_raw_754_p: ksads_ptsd_raw_770_p |  |
| Family income | demo_comb_income_v2 | Combined household income |
| Parental education | demo_prnt_ed_v2 | Highest level of education received by parent |
| Race/ethnicity | race_ethnicity | 5 levels (Black, White, Hispanic, Asian, Other) |
| Body mass index (BMI) | BMI variable is not available in ABCD so we used anthroheightcalc (in inches) and anthroweightcalc (in pounds) | BMI was manually calculated using the following equation: bmi = 703* anthroweightcalc/(anthroheightcalc)^2^. We removed improbable values prior to analysis, which included five participants with impossible height and weight values (e.g., weight of 11lbs at age 10). |
| Participant age | Interview_age | Participant age at time of interview was originally given in months but was converted to years by dividing by 12. |

Table S1: ABCD variables used in current project.

### Trauma exposure data distribution
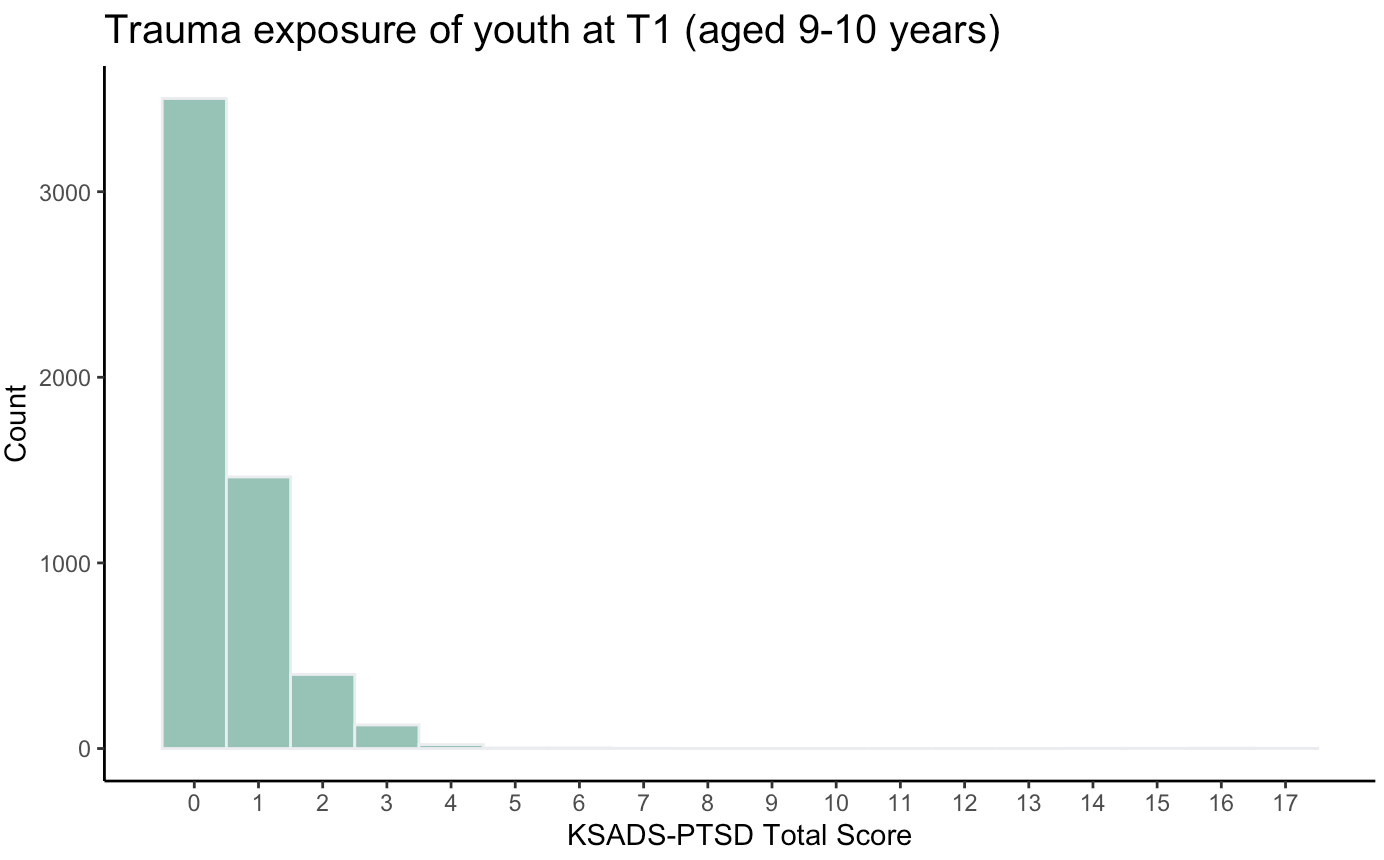


Figure S1: Histogram illustrating the distribution of the trauma variable (KSADS-PTSD subscale) in ABCD at T1 when youth are aged 9-10 years. KSADS-PTSD: Parent-report post-traumatic stress disorder subscale from the Kiddie Schedule for Affective Disorders and Schizophrenia for DSM-5.


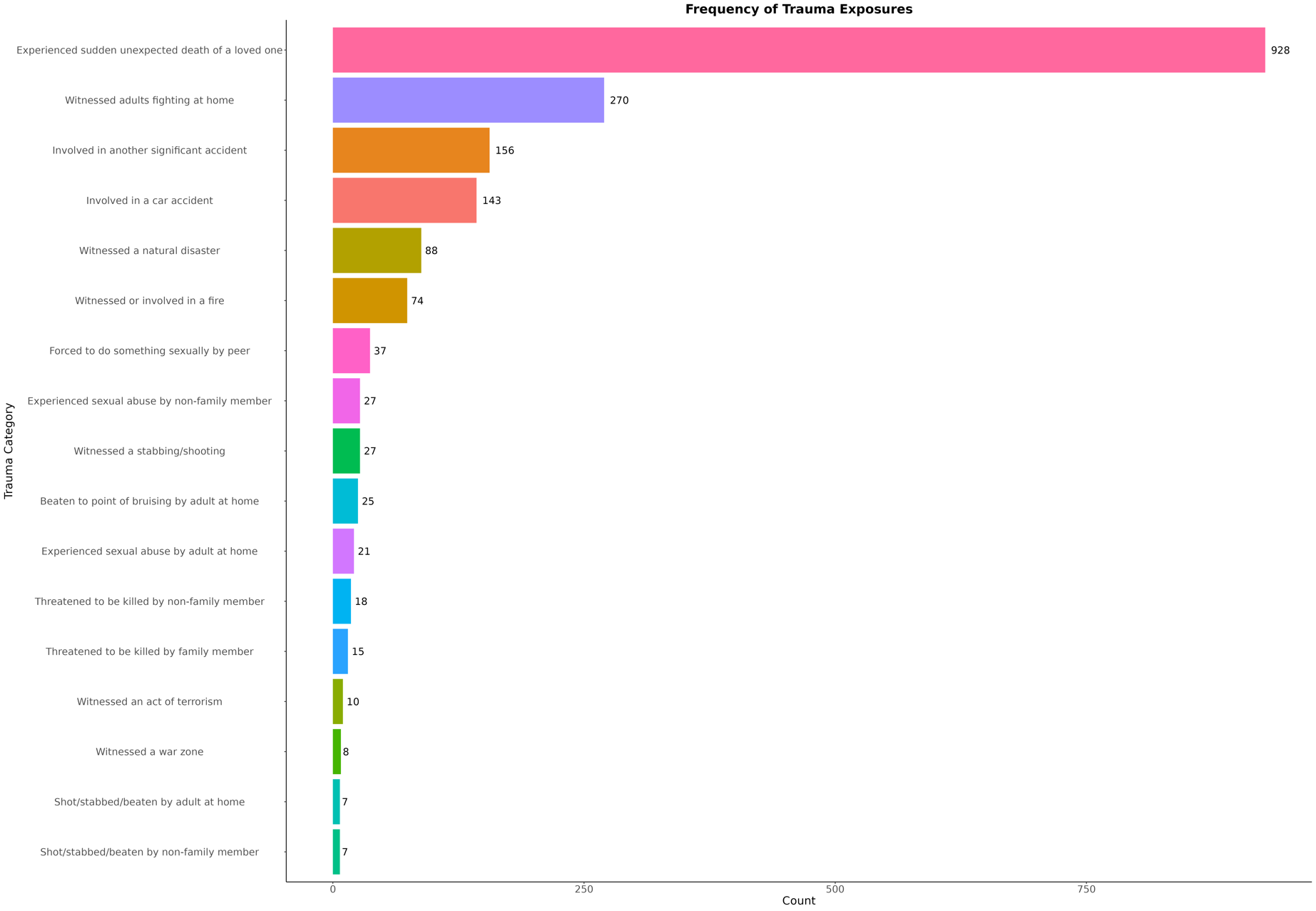


Figure S2. Frequency of the type of traumatic events reported at T1 (baseline) based on the KSADS-PTSD: Parent-report post-traumatic stress disorder subscale from the Kiddie Schedule for Affective Disorders and Schizophrenia for DSM-5.

| **No. of traumatic events experienced (%)** | **0** | **1** | **2** | **3** | **4** | **5** | **6** | **7** | **8** | **9** | **10** | **11** | **12** | **13** | **14** | **15** | **16** | **17** |
| --- | --- | --- | --- | --- | --- | --- | --- | --- | --- | --- | --- | --- | --- | --- | --- | --- | --- | --- |
| Early starters | 127 (53.09%) | 103 (31.79%) | 37 (11.42%) | 8 (2.47%) | 3 (0.93%) | 0 (0%) | 0 (0%) | 1 (0.31%) | 0 (0%) | 0 (0%) | 0 (0%) | 0 (0%) | 0 (0%) | 0 (0%) | 0 (0%) | 0 (0%) | 0 (0%) | 0 (0%) |
| Typical Developers | 1784 (63.31%) | 763 (27.08%) | 201 (7.13%) | 53 (1.88%) | 13 (0.46%) | 1 (0.04%) | 2 (0.07%) | 0 (0%) | 0 (0%) | 0 (0%) | 0 (0%) | 0 (0%) | 0 (0%) | 0 (0%) | 0 (0%) | 0 (0%) | 0 (0%) | 1 (0.04%) |
| Slow  developers | 360 (68.4%) | 130 (24.71%) | 22 (4.18%) | 12 (2.28%) | 0 (0%) | 1 (0.19%) | 0 (0%) | 0 (0%) | 0 (0%) | 0 (0%) | 0 (0%) | 0 (0%) | 0 (0%) | 0 (0%) | 0 (0%) | 0 (0%) | 1 (0.19%) | 0 (0%) |

Table S2. Number and percentage of traumatic events experienced for each puberty class. Traumatic events were measured using the parent-report post-traumatic stress disorder subscale from the Kiddie Schedule for Affective Disorders and Schizophrenia for DSM-5 (KSADS-PTSD).

### Puberty data scoring and distribution

The Pubertal Development Scale (PDS; Petersen et al., 1988) was used to examine the perceived development of secondary sex characteristics such as growth spurts, body hair growth, skin changes, breast development and menarche in girls, and voice changes and growth of facial hair in boys. The PDS includes five-items, and each characteristic is rated on a 4-point scale (1 = no development; 2 = development has barely begun; 3 = development is definitely underway; and 4 = development is complete; except menstruation, which is coded 1 = has not begun, 4 = has begun). Higher scores reflect more advanced pubertal maturation. We used average PDS scores from T1, T2, T3, and T4 as our measure of perceived pubertal maturation (see Figure S2). Items were considered missing if a response was left blank or answered, “I don’t know” or “refused to answer”. If participants had a missing value for any of the five items used to calculate the average PDS score, their average PDS score was coded as “missing”.


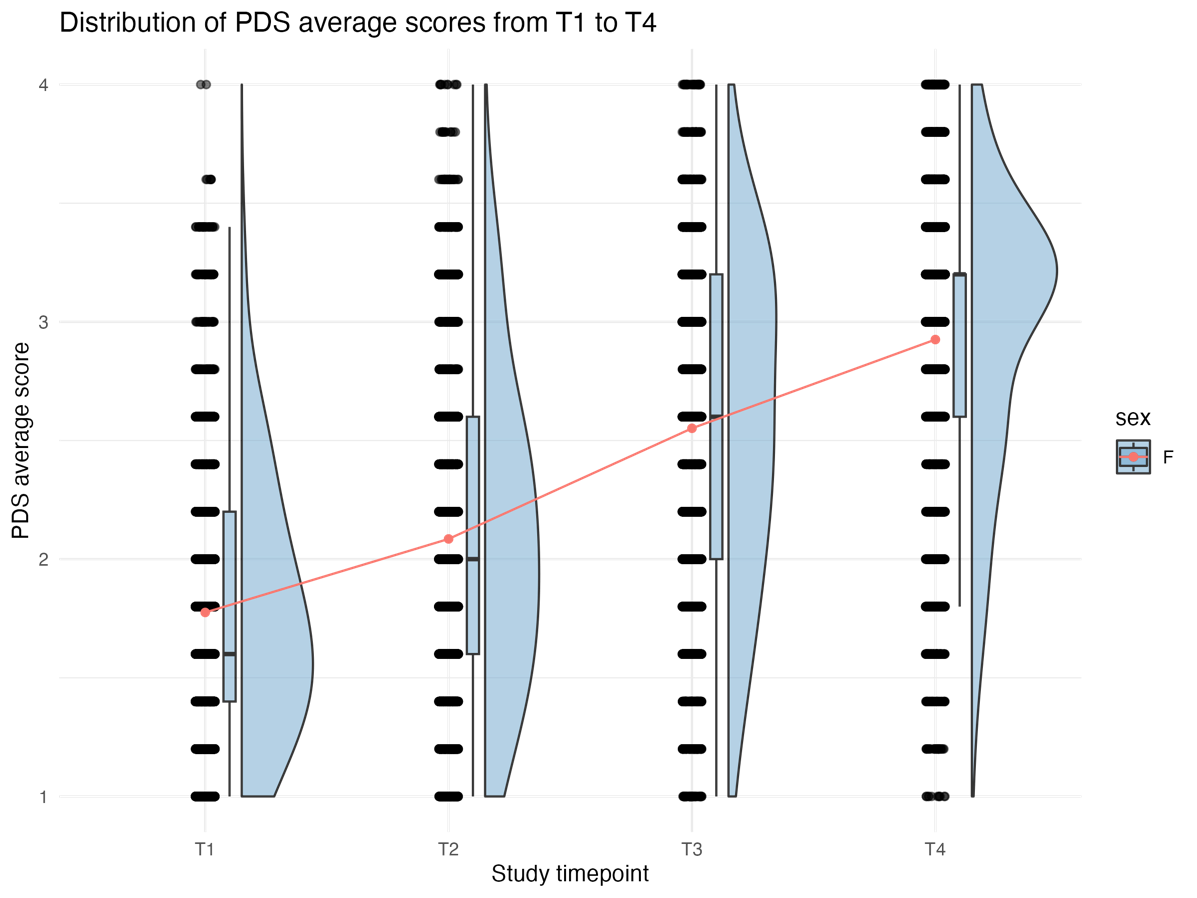


Figure S3: Distribution of Pubertal Development Scale (PDS) average scores across T1 (baseline) to T4 (three-year follow-up).

### Association between caregiver-report and youth-self report puberty measures

As outlined in the main text, the PDS caregiver-report was used in our main, pre-registered analyses. However, caregiver-report and youth self-report PDS scores were substantially correlated at each of the four timepoints (See Table S3) and increased over time. For completeness, we report correlations between caregiver-report and youth-self report for females and males in Table S3, although analyses in the male only sample was not pre-registered and are reported in full in the Section 6 of the SI. We note that the correlations between reports were stronger for females compared to males.

|  | **Females** |  | **Males** |  |
| --- | --- | --- | --- | --- |
| **Timepoint** | ***r*** | ***p*** | ***r*** | ***p*** |
| T1 | 0.56 | <0.001 | 0.25 | <0.001 |
| T2 | 0.74 | <0.001 | 0.43 | <0.001 |
| T3 | 0.80 | <0.001 | 0.60 | <0.001 |
| T4 | 0.76 | <0.001 | 0.68 | <0.001 |

Table S3. Pearson’s correlations between Pubertal Development Scale (PDS) caregiver report and youth self-report at T1 (baseline), T2 (one year follow-up), T3 (two-year follow-up), and T4 (three-year follow-up).

### Internalising difficulties data cleaning

#### Distribution of residuals for BPM ~ Trauma category linear model before and after transforming data


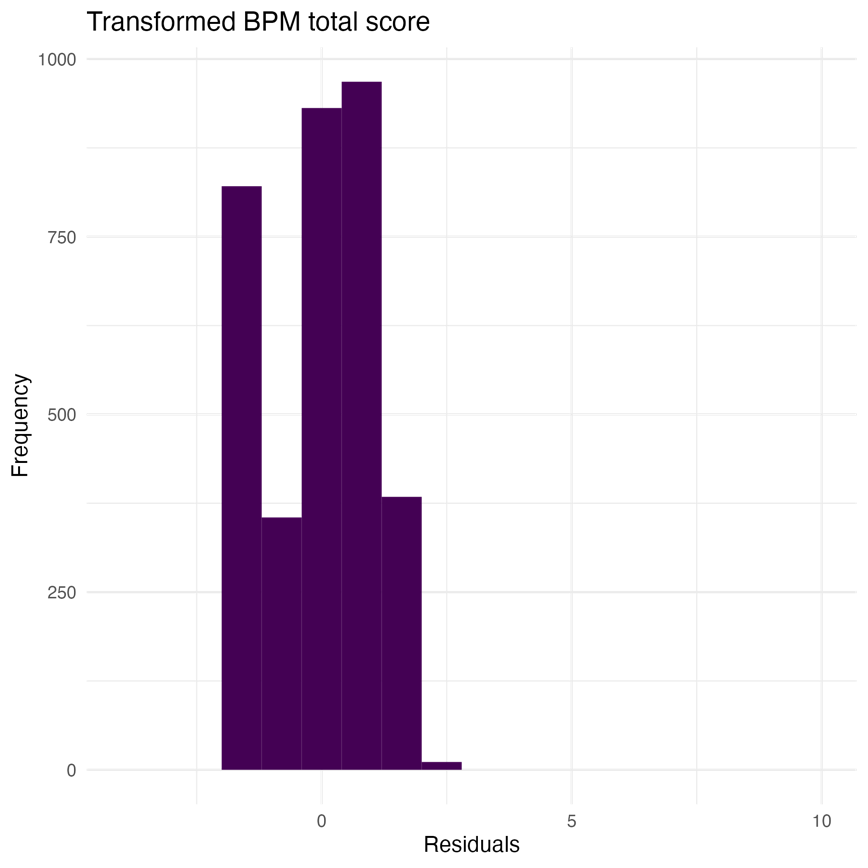

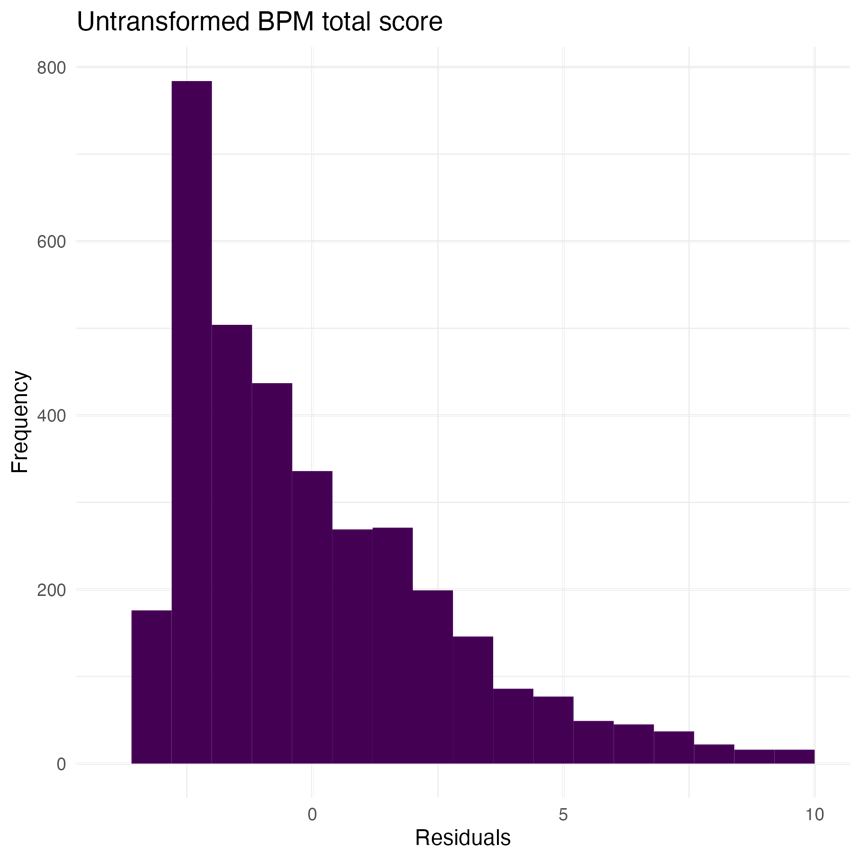


Figure S4: Untransformed (top) and transformed (bottom) (via Yeo-Johnson transformation method) BPM residuals.

## Section 2: Latent profile analysis results

### Latent profile analysis fit statistics with no within-class variance

|  | G | conv | loglik | npm | BIC | AIC | SABIC | entropy | ICL1 | ICL2 | %class1 | %class2 | %class3 | %class4 | %class5 | %class6 |
| --- | --- | --- | --- | --- | --- | --- | --- | --- | --- | --- | --- | --- | --- | --- | --- | --- |
| 1 class | 1 | 1 | -13000.68 | 3 | 26026.41 | 26007.36 | 26016.88 | 1 | 26026.41 | 26026.41 | 100 |  |  |  |  |  |
| 2 class | 2 | 1 | -10636.31 | 6 | 21322.7 | 21284.61 | 21303.64 | 0.76 | 22025.03 | 22057.59 | 55.86 | 44.14 |  |  |  |  |
| 3 class | 3 | 1 | -9628.53 | 9 | 19332.19 | 19275.06 | 19303.6 | 0.77 | 20378.28 | 20405.76 | 52 | 20.62 | 27.38 |  |  |  |
| 4 class | 4 | 1 | -9268.93 | 12 | 18638.05 | 18561.86 | 18599.92 | 0.74 | 20175.6 | 20245.44 | 12.19 | 29.94 | 16.54 | 41.33 |  |  |
| 5 class | 5 | 1 | -9159.85 | 15 | 18444.94 | 18349.71 | 18397.28 | 0.72 | 20319.61 | 20380.25 | 3.93 | 10.93 | 22.37 | 23.01 | 39.76 |  |
| 6 class | 6 | 1 | -9087.51 | 18 | 18325.3 | 18211.03 | 18268.11 | 0.7 | 20563.27 | 20697.34 | 2.46 | 20.02 | 38.41 | 13.33 | 7.03 | 18.75 |

Table S4: Summary fit statistics for classes 1 to 6 for latent profile analysis with no within-class variance. Notes: G = number of classes or profiles in the latent profile analysis; conv = convergence status, where 1 = success and 0 = model did not converge; loglik = log-likelihood of model; npm = number of parameters estimated in the model; BIC = Bayesian Information Criterion; AIC = Akaike Information Criterion; SABIC = Sample-size Adjusted Bayesian Information Criterion; ILC1 = Integrated Complete Likelihood Criterion 1; ILC2 = Integrated Complete Likelihood Criterion 2.

### Latent profile analysis fit statistics with within-class variance

|  | G | conv | loglik | npm | BIC | AIC | SABIC | entropy | ICL1 | ICL2 | %class1 | %class2 | %class3 | %class4 |
| --- | --- | --- | --- | --- | --- | --- | --- | --- | --- | --- | --- | --- | --- | --- |
| 3 class | 3 | 1 | -9010.95 | 14 | 18138.77 | 18049.89 | 18094.29 | 0.71 | 19500.82 | 19518.45 | 14.63 | 76.4 | 8.97 |  |
| 4 class | 4 | 1 | -8962.11 | 18 | 18074.5 | 17960.22 | 18017.3 | 0.73 | 19680.44 | 19742.96 | 16.31 | 67.64 | 15.57 | 0.47 |

Table S5. Latent profile analysis with within-class variance for a three and four class solution, which were the two best fitting models in the latent profile analysis with no within-class variance. Notes: G = number of classes or profiles in the latent profile analysis; conv = convergence status, where 1 = success and 0 = model did not converge; loglik = log-likelihood of model; npm = number of parameters estimated in the model; BIC = Bayesian Information Criterion; AIC = Akaike Information Criterion; SABIC = Sample-size Adjusted Bayesian Information Criterion; ILC1 = Integrated Complete Likelihood Criterion 1; ILC2 = Integrated Complete Likelihood Criterion 2.

Figure S5. Density plots showing the variance of pubertal status values (i.e., the individual-level intercept values derived from the latent profile analysis) (Panel A) and pubertal tempo values (the individual-level slope values derived from the latent profile analysis) (Panel B).

## Section 3: Mediation results

Correlation matrix of numeric variables used in our mediation analyses


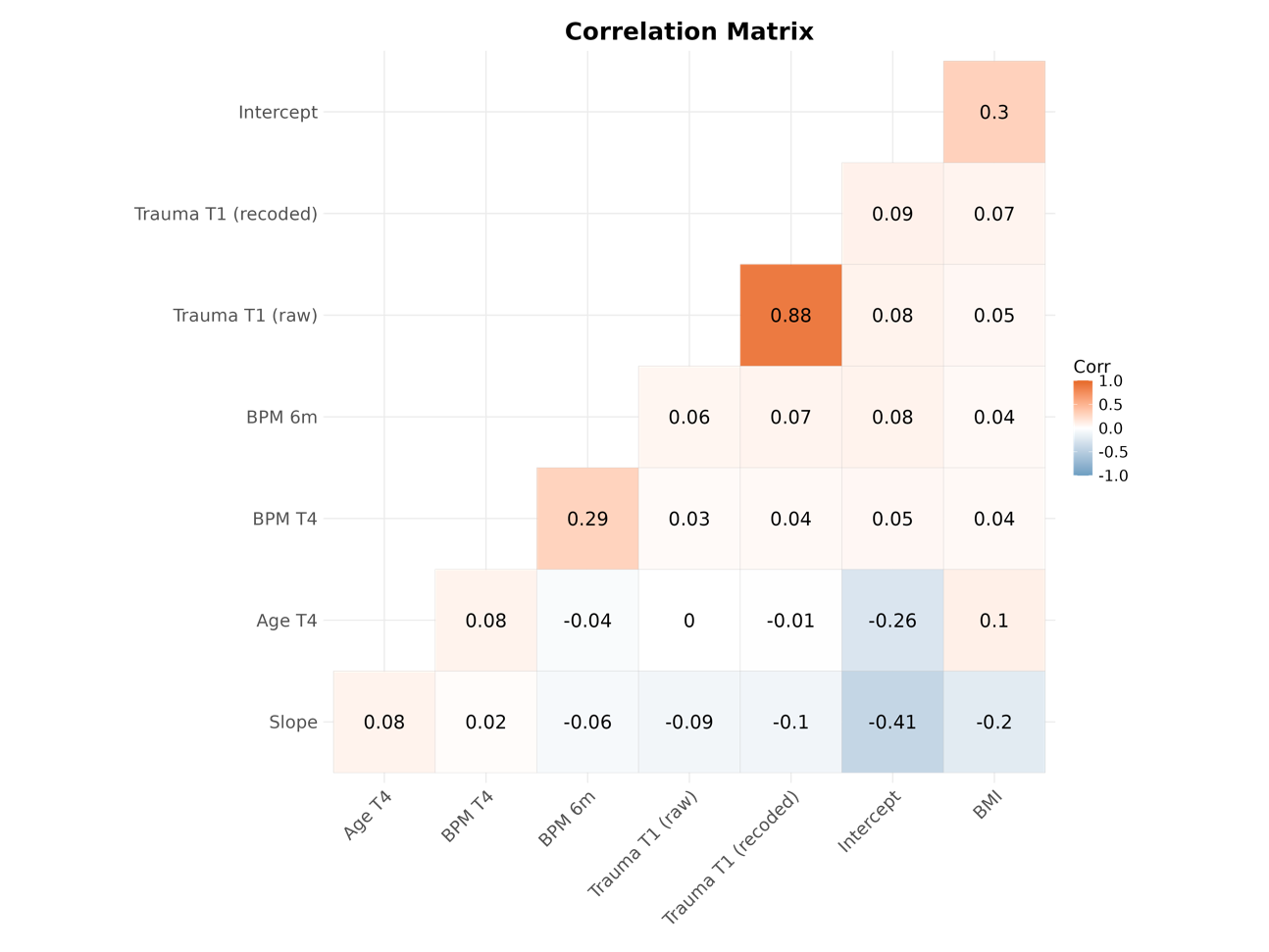


Figure S6. Correlation matrix of numeric variables used in mediation analyses. T1 = baseline timepoint; T4 = three-year follow up; 6m = six-month follow-up; BPM = Brief Problem Monitor, youth self-report measure of internalising problems; Intercept = individual-level intercept value extracted from the latent profile analysis; Slope = individual-level slope value extracted from the latent profile analysis; BMI = body-mass index; Trauma T1 (recoded) = recoded trauma variable with values ranging 0-2, which was used in our main mediation analyses; Trauma T1 (raw) = raw total sum score from the Kiddie Schedule for Affective Disorders and Schizophrenia for DSM-5 (KSADS-PTSD), which was used in our sensitivity analyses; Age T4 = age in years at three-year follow-up timepoint.

| lhs | op | rhs | label | Estimate (unstandardised) | Standard error | z | p.value | ci.lower | ci.upper | Standardised Estimate |
| --- | --- | --- | --- | --- | --- | --- | --- | --- | --- | --- |
| slope | ~ | trauma | a1 | -0.151 | 0.024 | -6.426 | 0 | -0.196 | -0.103 | -0.101 |
| slope | ~ | age |  | 0.128 | 0.026 | 4.883 | 0 | 0.077 | 0.18 | 0.082 |
| intercept | ~ | trauma | a2 | 0.148 | 0.024 | 6.278 | 0 | 0.102 | 0.194 | 0.098 |
| intercept | ~ | age |  | -0.395 | 0.025 | -15.59 | 0 | -0.443 | -0.344 | -0.252 |
| intSx | ~ | slope | b1 | 0.057 | 0.019 | 3.074 | 0.002 | 0.021 | 0.095 | 0.057 |
| intSx | ~ | intercept | b2 | 0.105 | 0.019 | 5.383 | 0 | 0.066 | 0.142 | 0.105 |
| intSx | ~ | trauma | c | 0.055 | 0.026 | 2.114 | 0.035 | 0.003 | 0.105 | 0.037 |
| intSx | ~ | age |  | 0.185 | 0.027 | 6.826 | 0 | 0.129 | 0.237 | 0.118 |
| slope | ~~ | intercept |  | -0.373 | 0.016 | -23.885 | 0 | -0.403 | -0.342 | -0.391 |
| slope | ~~ | slope |  | 0.983 | 0.028 | 35.589 | 0 | 0.933 | 1.041 | 0.983 |
| intercept | ~~ | intercept |  | 0.926 | 0.02 | 45.186 | 0 | 0.887 | 0.968 | 0.926 |
| intSx | ~~ | intSx |  | 0.98 | 0.016 | 60.563 | 0 | 0.95 | 1.013 | 0.98 |
| trauma | ~~ | trauma |  | 0.443 | 0.01 | 42.474 | 0 | 0.423 | 0.464 | 1 |
| trauma | ~~ | age |  | -0.004 | 0.007 | -0.544 | 0.586 | -0.017 | 0.01 | -0.009 |
| age | ~~ | age |  | 0.409 | 0.007 | 60.964 | 0 | 0.396 | 0.422 | 1 |
| slope | ~1 |  |  | -0.161 | 0.087 | -1.854 | 0.064 | -0.328 | 0.013 | -0.161 |
| intercept | ~1 |  |  | 0.963 | 0.082 | 11.723 | 0 | 0.797 | 1.118 | 0.963 |
| intSx | ~1 |  |  | -0.633 | 0.091 | -6.945 | 0 | -0.808 | -0.454 | -0.633 |
| trauma | ~1 |  |  | 1.466 | 0.011 | 134.396 | 0 | 1.445 | 1.488 | 2.202 |
| age | ~1 |  |  | 2.987 | 0.011 | 281.172 | 0 | 2.966 | 3.008 | 4.671 |
| Indirect 1 (slope as mediator) | := | a1*b1 | Indirect 1 (slope as mediator) | -0.009 | 0.003 | -2.807 | 0.005 | -0.015 | -0.003 | -0.006 |
| indirect2 (intercept as mediator) | := | a2*b2 | Indirect 1 (intercept as mediator) | 0.015 | 0.004 | 4.005 | 0 | 0.009 | 0.024 | 0.010 |
| Total effect | := | c+(a1*b1)+(a2*b2) | total | 0.062 | 0.026 | 2.37 | 0.018 | 0.01 | 0.114 | 0.041 |
| Direct effect | := | c | direct | 0.055 | 0.026 | 2.114 | 0.035 | 0.003 | 0.105 | 0.037 |

Table S6. Lavaan output for the multiple mediation model. Notes on model syntax: lhs = left hand side of equation; op = operator; rhs = right hand side of equation; label = mediation path label; ci = 95% confidence interval. IntSx = internalising symptoms. Notes on operators: ~ = regression, ~~ covariance, := specified parameters.

### Intercept stand-alone mediation model

Figure S7: Intercept-only model where pubertal status at baseline (individual-level intercept) was included as the mediator, trauma exposure was the predictor, and internalising symptoms was the outcome. * = significant at p ≤0.05, ** = significant at p ≤ 0.01, *** = significant at p ≤0.001. Beta values reported are standardised. Note: For clarity of plotting, the covariate of age was not included in the figure.

| lhs | op | rhs | label | Estimate (unstandardised) | Standard error | z | p.value | ci.lower | ci.upper | Standardised Estimate |
| --- | --- | --- | --- | --- | --- | --- | --- | --- | --- | --- |
| intercept | ~ | trauma | a1 | 0.148 | 0.024 | 6.278 | 0 | 0.102 | 0.194 | 0.098 |
| intercept | ~ | age |  | -0.395 | 0.025 | -15.59 | 0 | -0.443 | -0.344 | -0.252 |
| intSx | ~ | intercept | b1 | 0.082 | 0.018 | 4.517 | 0 | 0.046 | 0.117 | 0.082 |
| intSx | ~ | trauma | c | 0.049 | 0.026 | 1.897 | 0.058 | -0.002 | 0.1 | 0.033 |
| intSx | ~ | age |  | 0.183 | 0.027 | 6.756 | 0 | 0.127 | 0.235 | 0.117 |
| intercept | ~~ | intercept |  | 0.926 | 0.02 | 45.186 | 0 | 0.887 | 0.968 | 0.926 |
| intSx | ~~ | intSx |  | 0.983 | 0.016 | 60.755 | 0 | 0.952 | 1.015 | 0.983 |
| trauma | ~~ | trauma |  | 0.443 | 0 | 0 | 0 | 0.443 | 0.443 | 1 |
| trauma | ~~ | age |  | -0.004 | 0 | 0 | 0 | -0.004 | -0.004 | -0.009 |
| age | ~~ | age |  | 0.409 | 0 | 0 | 0 | 0.409 | 0.409 | 1 |
| intercept | ~1 |  |  | 0.963 | 0.082 | 11.723 | 0 | 0.797 | 1.118 | 0.963 |
| intSx | ~1 |  |  | -0.619 | 0.091 | -6.792 | 0 | -0.795 | -0.44 | -0.619 |
| trauma | ~1 |  |  | 1.466 | 0 | 0 | 0 | 1.466 | 1.466 | 2.202 |
| age | ~1 |  |  | 2.987 | 0 | 0 | 0 | 2.987 | 2.987 | 4.671 |
| Indirect (intercept as mediator) | := | a1*b1 | indirect1 | 0.012 | 0.003 | 3.604 | 0 | 0.006 | 0.02 | 0.008 |
| Total effect | := | c+(a1*b1) | total | 0.062 | 0.026 | 2.352 | 0.019 | 0.01 | 0.113 | 0.041 |
| Direct effect | := | c | direct | 0.049 | 0.026 | 1.897 | 0.058 | -0.002 | 0.1 | 0.033 |

Table S7. Lavaan output for the intercept-only mediation model. Notes on model syntax: lhs = left hand side of equation; op = operator; rhs = right hand side of equation; label = mediation path label; ci = 95% confidence interval. IntSx = internalising symptoms. Notes on operators: ~ = regression, ~~ covariance; := specified parameters.

### Slope stand-alone mediation model

Figure S8: Slope-only model where pubertal tempo (individual-level slope) was included as the mediator, trauma exposure was the predictor, and internalising symptoms was the outcome. * = significant at p ≤0.05, ** = significant at p ≤ 0.01, *** = significant at p ≤0.001. Beta values reported are standardised. Note: For clarity of plotting, the covariate of age was not included in the figure.

| lhs | op | rhs | label | Estimate (unstandardised) | Standard error | z | p.value | ci.lower | ci.upper | Standardised Estimate |
| --- | --- | --- | --- | --- | --- | --- | --- | --- | --- | --- |
| slope | ~ | trauma | a1 | -0.151 | 0.024 | -6.425 | 0 | -0.196 | -0.103 | -0.101 |
| slope | ~ | age |  | 0.128 | 0.026 | 4.883 | 0 | 0.077 | 0.18 | 0.082 |
| intSx | ~ | slope | b1 | 0.018 | 0.017 | 1.032 | 0.302 | -0.014 | 0.053 | 0.018 |
| intSx | ~ | trauma | c | 0.063 | 0.026 | 2.401 | 0.016 | 0.011 | 0.115 | 0.042 |
| intSx | ~ | age |  | 0.149 | 0.026 | 5.66 | 0 | 0.095 | 0.198 | 0.095 |
| slope | ~~ | slope |  | 0.983 | 0.028 | 35.589 | 0 | 0.933 | 1.041 | 0.983 |
| intSx | ~~ | intSx |  | 0.988 | 0.016 | 61.137 | 0 | 0.958 | 1.022 | 0.989 |
| trauma | ~~ | trauma |  | 0.443 | 0 | 0 | 0 | 0.443 | 0.443 | 1 |
| trauma | ~~ | age |  | -0.004 | 0 | 0 | 0 | -0.004 | -0.004 | -0.009 |
| age | ~~ | age |  | 0.409 | 0 | 0 | 0 | 0.409 | 0.409 | 1 |
| slope | ~1 |  |  | -0.161 | 0.087 | -1.854 | 0.064 | -0.328 | 0.013 | -0.161 |
| intSx | ~1 |  |  | -0.539 | 0.09 | -6.004 | 0 | -0.715 | -0.364 | -0.539 |
| trauma | ~1 |  |  | 1.466 | 0 | 0 | 0 | 1.466 | 1.466 | 2.202 |
| age | ~1 |  |  | 2.987 | 0 | 0 | 0 | 2.987 | 2.987 | 4.671 |
| indirect1  (slope as mediator) | := | a1*b1 | indirect1 | -0.003 | 0.003 | -1.019 | 0.308 | -0.008 | 0.002 | -0.002 |
| Total effect | := | c+(a1*b1) | total | 0.061 | 0.026 | 2.315 | 0.021 | 0.009 | 0.112 | 0.04 |
| Direct effect | := | c | direct | 0.063 | 0.026 | 2.4 | 0.016 | 0.011 | 0.115 | 0.042 |

Table S8. Lavaan output for the slope-only mediation model. Notes on model syntax: lhs = left hand side of equation; op = operator; rhs = right hand side of equation; label = mediation path label; ci = 95% confidence interval. IntSx = internalising symptoms. Notes on operators: ~ = regression, ~~ covariance; := specified parameters.

### Section 4: Sensitivity analyses

### Additional covariates

When we included participant body mass index (BMI) and household income as fixed covariates in our multiple mediation model our effects attenuated slightly but remained significant — direct effect: ß = 0.050, p = 0.040, 95% CI [0.004, 0.105]; indirect effects (intercept mediator: ß = 0.009, p = 0.002, 95% CI [0.004, 0.016], slope mediator: ß = -0.006, p = 0.010, 95% CI [-0.011, -0.002]). When we added race/ethnicity (recoded as a dummy variable) to this mediation model, the direct effect was no longer significant (direct effect: ß = 0.038, p = 0.10, 95% CI [-0.007, 0.087]). However, the indirect effects for both the intercept as mediator (ß = 0.008, p = 0.008, 95% CI [0.003, 0.014] and the slope as mediator (ß = -0.004, p = 0.038, 95% CI [-0.009, -0.001]) remained significant.

When earlier internalising symptoms, measured at the six-month follow-up, were accounted for in our multiple mediation model, the indirect effects persisted (intercept mediator: ß = 0.013, p <0.001, 95% CI [0.007, 0.021], slope mediator: ß = -0.010, p = 0.002, 95% CI [-0.016, -0.004] but the direct effect between trauma exposure and internalising symptoms was no longer significant (ß = 0.028, p = 0.259, 95% CI [-0.022, 0.077]. We note that we would have ideally accounted for earlier internalising symptoms at the same timepoint at which trauma exposure was measured (i.e., at baseline) but the six-month follow-up is the first timepoint at which youth-self reported internalising symptoms is available in the ABCD Study.

We also re-ran our main mediation analysis using the original KSADS PTSD sum score trauma variable as our predictor (range = 0 to 17). Results were consistent with our main model for both the direct effect (ß = 0.06, p = 0.03, 95% CI [0.006, 0.106]) and the indirect effects (intercept mediator: ß = 0.015, p ≤ 0.001, 95% CI [0.009, 0.024], slope mediator: ß = -0.009, p = 0.005, 95% CI [-0.016, -0.003]).

To assess whether a significant portion of variance could be attributed to site differences, we calculated the intraclass correlation coefficient (ICC) for site in each path of our mediation model. We used linear mixed effects models to do this (via the *lmer* function in R) and calculated the ICC using the *icc* function from the *performance* package. For path c (internalising symptoms ~ trauma exposure + age + 1| site), ICC for site variable = 0.007. For path a (PDS average score  ~ trauma exposure + age + 1| site), ICC for site variable = 0.023. For path b (PDS average ~ internalising symptoms + age + 1| site), ICC for site variable = 0.006. ICC values were close to zero, suggesting that site differences did not contribute a substantial amount of variance. Thus, we did not include site as a covariate in our mediation models in the interest of model parsimony.

## Section 5: Exploratory analyses

### The timing of pubertal timing

##### Calculation of pubertal timing measure

In line with existing research, pubertal timing was calculated for each participant by regressing their average PDS score on their age using a linear mixed-effects model with subject ID and site as random effects (MacSweeney et al., 2023; Vijayakumar et al., 2023). We then used the model residuals as a measure of pubertal timing. This score represents the residual differences for each participant at each specific time point. Here, pubertal timing is defined as an individual’s pubertal development relative to their same-age, same-sex peers (Mendle et al., 2019).

##### Exploratory data analysis

The aim of exploratory analysis was to examine whether the timing of pubertal timing mediated the association between prior trauma exposure and later internalising difficulties. We first separated our sample into 6-month age bins using the age range 9-14 years (i.e., 9–9.5-year-olds, 9.5–10-year-olds, 10–10.5-year-olds …. 13.5–14-year-olds), which totalled 10 subgroups. 6-month intervals were chosen instead of 12-month intervals to avoid having repeated measures from participants within the same age group, as the interval between annual assessments was less than 12 months for some participants. Participants that were aged below age 9 years at the T1 or above age 14 years at T4 were excluded from the analysis (N = 144). We found that the interval between yearly study visits in ABCD was less than 6 months for 25 participants. In these instances, we randomly selected one observation per participant per age bin. This ensured that there were no repeated measures for an individual participant within each age bin.

Next, for each age-bin separately, we ran mediation analyses using *lavaan* to test whether pubertal timing mediated the association between trauma exposure and later internalising difficulties. After correction for multiple comparisons, we did not find any evidence for pubertal timing as a mediator between trauma exposure and later depression as indicated by the absence of a significant indirect effect. Our findings are reported in full in Table S4.

| age group | N | effect | Standardised estimate | Standard error | z | pvalue | ci.lower | ci.upper |
| --- | --- | --- | --- | --- | --- | --- | --- | --- |
|  |  | indirect | 0 | 0 | -0.39 | 0.7 | -0.01 | 0.01 |
| 9- to 9.5-year-olds | 1198 | total | 0.08 | 0.05 | 1.65 | 0.1 | -0.02 | 0.17 |
|  |  | direct effect | 0.08 | 0.05 | 1.69 | 0.09 | -0.02 | 0.17 |
| 9.5- to 10-year-olds | 1066 | indirect | 0 | 0 | 0.14 | 0.89 | 0 | 0.01 |
|  |  | total | 0.06 | 0.05 | 1.2 | 0.23 | -0.04 | 0.15 |
|  |  | direct effect | 0.06 | 0.05 | 1.19 | 0.23 | -0.04 | 0.15 |
| 10- to 10.5-year-olds | 1956 | indirect | 0 | 0 | -0.16 | 0.87 | 0 | 0 |
|  |  | total | 0.07 | 0.04 | 1.94 | 0.05 | 0 | 0.15 |
|  |  | direct effect | 0.07 | 0.04 | 1.94 | 0.05 | 0 | 0.15 |
| 10.5- to 11-year-olds | 1919 | indirect | 0 | 0 | -0.25 | 0.8 | -0.01 | 0 |
|  |  | total | 0.12 | 0.04 | 3.03 | 0 | 0.04 | 0.19 |
|  |  | direct effect | 0.12 | 0.04 | 3.04 | 0 | 0.04 | 0.19 |
| 11- to 11.5-year-olds | 1771 | indirect | 0 | 0 | -0.25 | 0.8 | -0.01 | 0 |
|  |  | total | 0.08 | 0.04 | 2.19 | 0.03 | 0.01 | 0.15 |
|  |  | direct effect | 0.08 | 0.04 | 2.2 | 0.03 | 0.01 | 0.15 |
| 11.5- to 12-year-olds | 1920 | indirect | 0 | 0 | 0.9 | 0.37 | 0 | 0.01 |
|  |  | total | 0.07 | 0.04 | 1.81 | 0.07 | -0.01 | 0.15 |
|  |  | direct effect | 0.07 | 0.04 | 1.76 | 0.08 | -0.01 | 0.15 |
| 12- to 12.5-year-olds | 1953 | indirect | 0 | 0 | -0.32 | 0.75 | 0 | 0 |
|  |  | total | 0.1 | 0.04 | 2.86 | 0 | 0.03 | 0.17 |
|  |  | direct effect | 0.1 | 0.04 | 2.87 | 0 | 0.03 | 0.17 |
| 12.5- to 13-year-olds | 1631 | indirect | 0 | 0 | -0.26 | 0.79 | -0.01 | 0 |
|  |  | total | 0.05 | 0.04 | 1.16 | 0.24 | -0.03 | 0.13 |
|  |  | direct effect | 0.05 | 0.04 | 1.17 | 0.24 | -0.03 | 0.13 |
| 13- to 13.5-year-olds | 1121 | indirect | 0 | 0 | 0.03 | 0.97 | 0 | 0 |
|  |  | total | 0.11 | 0.05 | 2.06 | 0.04 | 0.01 | 0.22 |
|  |  | direct effect | 0.1 | 0.05 | 2.05 | 0.04 | 0.01 | 0.22 |
| 13.5- to 14-year-olds | 781 | indirect | 0 | 0 | -0.27 | 0.79 | -0.01 | 0 |
|  |  | total | 0.03 | 0.07 | 0.39 | 0.7 | -0.1 | 0.16 |
|  |  | direct effect | 0.03 | 0.07 | 0.4 | 0.69 | -0.1 | 0.16 |

Table S9: Indirect, total, and direct effects for our exploratory mediation model where trauma exposure = predictor, pubertal timing = mediator, and internalising symptoms = the outcome. No associations remained significant after correction for multiple comparisons. The direct effect reported is between trauma exposure and internalising symptoms.

## Section 6: Male sample analysis

Although the primary aim of our pre-registered study was the examine the relationship between trauma exposure, pubertal development, and internalising symptoms in female youth, for completeness, and at the request of reviewers, we re-ran our main analyses in males. As our main hypotheses did not pertain to testing sex-differences and due to the significant differences in the degree of pubertal development in the available age-range from ABCD (Figure S9) we do not make a direct comparison between the male and female results.

#### Male results

We report the characteristics of the male sample in Table S12.

#### Latent profiles of pubertal development

The summary fit statistics for the latent profile analysis with no within-class variance are presented in Table S14. Fit statistics (e.g., BIC, AIC, entropy) suggested that the 3- and 4-class solutions were the optimal models. As per the approach taken in our main analysis, we ran these class solutions with within-class variance. Like the female results, a 3-class solution was found to be the best model fit across criteria. The class percentages and mean values for the intercept and slope for the 3-class solution are given in Table S13. See Table S15 for the full solutions for the 3-class and 4-class models.

Like our main analyses in females, our results demonstrated three distinct patters of pubertal development (Figure S12): “typical developers”, “slow developers”, and “early starters”. Although the class membership percentages differed between males and females, we used the same class labels given that we observed similar patterns of pubertal development between the sexes even though the males were less advanced in puberty overall, which is to be expected in this age range. Typical developers (51% of the sample) were characterised by low intercept values (mean PDS = 1.33) suggesting that they were pre-pubertal/early in puberty at T1 but exhibited the most rapid pace of development over time (shown by the highest slope value) such that they were midway through puberty at T4. Slow developers (44% of sample) exhibited similar low intercept values at T1 (mean PDS = 1.29) but had a lower slope value than the typical developers, such that they were still in the early stages of puberty at T4. Finally, early starters (5% of the sample) demonstrated more advanced pubertal maturation (mean PDS = 2.03) such that they were around midway through puberty at T1 but then showed a slower pace of development over time (i.e., lower slope value) compared to the typical developers.

We note that the percentage of individuals in the “slow developers” class was considerably larger in males compared to females. This difference is likely a reflection of the significant number of males whose “pubertal development had barely begun” (as per the PDS item response). Thus, this class of males is likely better described as “slower developers” rather than “slow developers” as we do not yet have the longitudinal data needed to determine whether their development lags behind that of the typical developers over time or if they “catch up”. However, for ease of interpretation, we use the same class labels for males and females.


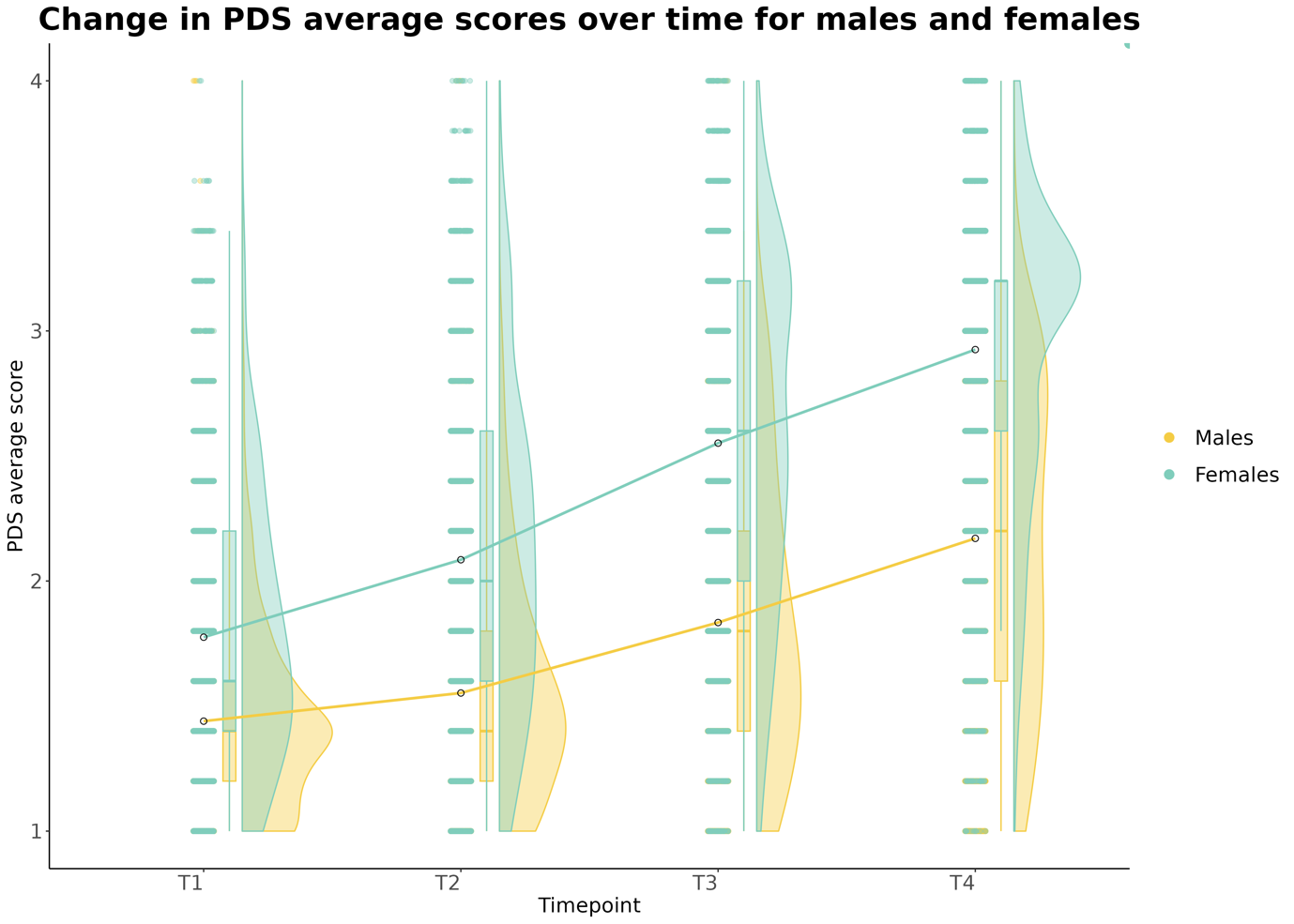


Figure S9. Change in Pubertal Development Scale (PDS) average scores over time for males and females from T1 (baseline) to T4 (three-year follow-up).

#### Male sample: Puberty class membership, trauma exposure, and internalising symptoms

Like our main analyses, a statistically significant difference was found in the degree of trauma exposure between puberty development classes: *H*(2) = 13.6, p = 0.001 (Figure S10). Dunn’s posthoc tests (with Bonferroni correction) demonstrated that slow developers had significantly lower trauma exposure compared to typical developers (*p =* 0.001). However, in contrast to our main analyses in females, early starters did not show significantly higher levels of trauma exposure compared to typical developers (*p* = 0.59) or slow developers (*p* = 0.04) (See Table S10). Moreover, internalising symptoms were not found to differ significantly between the three puberty classes (*H*(2) = 2.58, p = 0.27) (see Table S10).


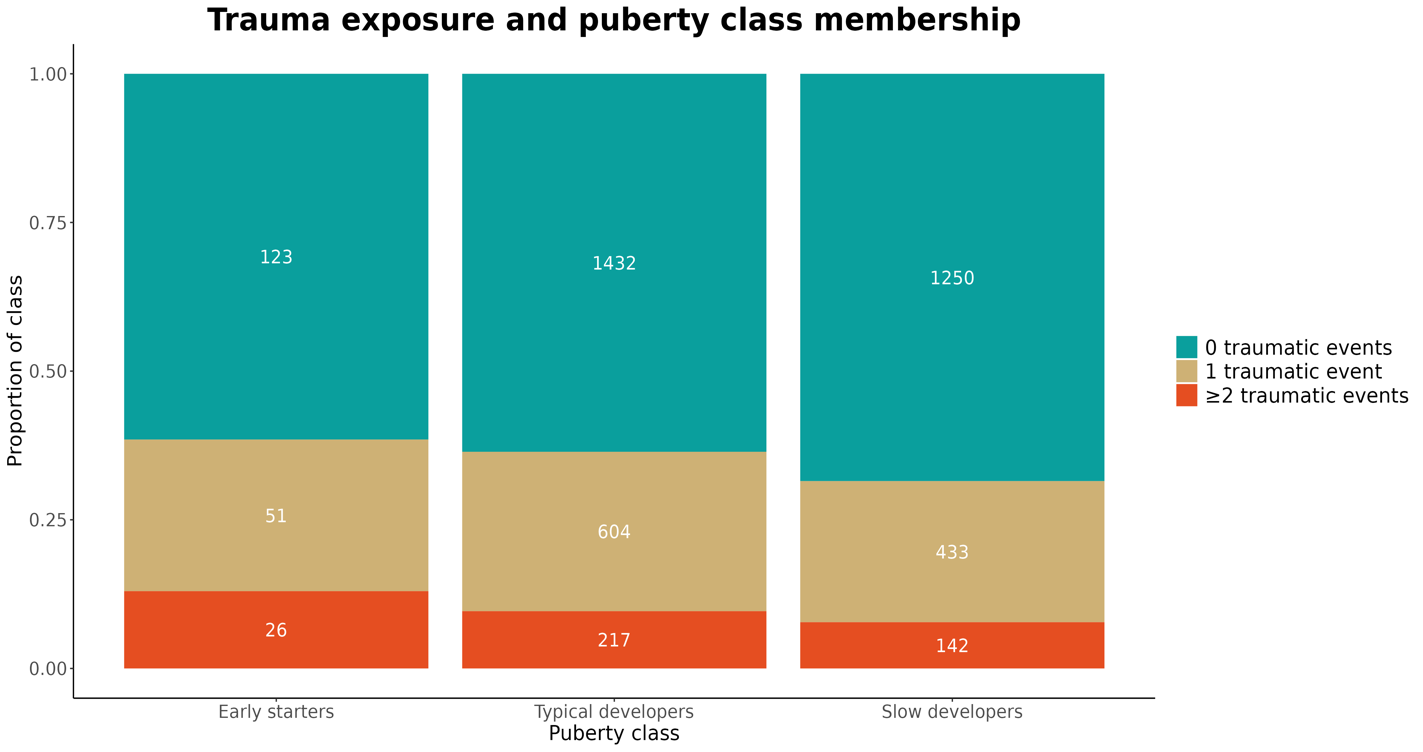


Figure S10. Male sample: Proportion and frequency counts of male participants with 0, 1 or ≥2 traumatic events reported in childhood, per puberty development class. N = 4278

|  | *Trauma Exposure* | | | *Internalising symptoms* | | |
| --- | --- | --- | --- | --- | --- | --- |
| **Class** | **N** | **Mean** | **SD** | **N** | **Mean** | **SD** |
| Early starters | 200 | 1.52 | 0.71 | 184 | 1.6 | 2.08 |
| Typical developers | 2253 | 1.46 | 0.66 | 2140 | 1.54 | 2.01 |
| Slow developers | 1825 | 1.39 | 0.63 | 1719 | 1.47 | 1.99 |

Table S10. Male sample: Sample size, mean trauma exposure and internalising symptoms, and standard deviations (SD) per puberty development class. Note: Sample sizes vary due to missing internalising symptoms data.

##### Does puberty status or tempo mediate the association between childhood trauma exposure and later internalising symptoms in male youth?

Structural equation modelling was used to test whether pubertal status at ages 9–10 and tempo over ages 9–14, measured using individual-level intercept and slope values, derived from the latent profile analysis, mediated the relationship between childhood trauma exposure and later internalising symptoms at ages 13-14. The model was just identified, having zero degrees of freedom, so we do not report model fit statistics. Like our analyses in females, we found a significant direct effect, which suggests that greater trauma exposure in childhood was associated with a higher level of internalising symptoms (ß = 0.080, *p* = 0.001, 95% CI [0.031, 0.129]). Although a higher initial pubertal status (i.e., a greater value of pubertal status) mediated the positive association between trauma exposure and internalising symptoms, the degree of significance was marginal and the effect size was tiny (indirect effect: ß = 0.005, *p* =0.045, 95% CI [0.001, 0.010]; see Figure S11).Unlike our main analysis, we did not observe a significant indirect effect of pubertal tempo on the association between trauma exposure and internalising symptoms (indirect effect: ß = 0.000, *p* = 0.956, 95% CI [-0.001, 0.001]; Figure S11). See Table S11 for full model output.

Figure S11. Male sample: Multiple mediation model where individual-level intercept (pubertal status at T1) and slope (pubertal tempo from T1 to T4) values were included as mediators, trauma exposure was the predictor, and internalising symptoms was the outcome at T4. Standardised regression coefficients are shown for each path. The residual variance of the mediator and outcome variables, indicated by the double-headed curved arrows, reflects the variability not explained by the model. The double-headed curved arrow between pubertal status and tempo reflects the correlation between the residuals of these two variables. Indirect standardised effects: Pubertal status (intercept): ß = 0.005 (i.e., 0.12×0.04), Pubertal tempo: ß = 0.000 (i.e., 0.01×0.00). * = significant at p ≤0.05, ** = significant at p ≤ 0.01, *** = significant at p ≤0.001. Note: For clarity of plotting, the covariate of age was not included in the figure, but full details of the mediation model can be found in the Table S11.

| lhs | op | rhs | label | Estimate (unstandardised) | Standard error | z | p.value | ci.lower | ci.upper | Standardised Estimate |
| --- | --- | --- | --- | --- | --- | --- | --- | --- | --- | --- |
| slope | ~ | trauma | a1 | 0.013 | 0.023 | 0.553 | 0.58 | -0.033 | 0.058 | 0.008 |
| slope | ~ | age |  | 0.186 | 0.022 | 8.357 | 0 | 0.143 | 0.231 | 0.12 |
| intercept | ~ | trauma | a2 | 0.119 | 0.023 | 5.207 | 0 | 0.075 | 0.164 | 0.077 |
| intercept | ~ | age |  | -0.316 | 0.024 | -13.386 | 0 | -0.362 | -0.27 | -0.204 |
| intSx | ~ | slope | b1 | 0.002 | 0.017 | 0.115 | 0.909 | -0.032 | 0.035 | 0.002 |
| intSx | ~ | intercept | b2 | 0.039 | 0.018 | 2.22 | 0.026 | 0.005 | 0.074 | 0.039 |
| intSx | ~ | trauma | c | 0.08 | 0.025 | 3.221 | 0.001 | 0.031 | 0.13 | 0.052 |
| intSx | ~ | age |  | 0.061 | 0.026 | 2.353 | 0.019 | 0.01 | 0.112 | 0.04 |
| slope | ~~ | intercept |  | 0.365 | 0.025 | 14.579 | 0 | 0.313 | 0.413 | 0.377 |
| slope | ~~ | slope |  | 0.985 | 0.055 | 17.972 | 0 | 0.89 | 1.106 | 0.986 |
| intercept | ~~ | intercept |  | 0.953 | 0.039 | 24.13 | 0 | 0.886 | 1.042 | 0.953 |
| intSx | ~~ | intSx |  | 0.994 | 0.013 | 75.812 | 0 | 0.97 | 1.021 | 0.994 |
| trauma | ~~ | trauma |  | 0.426 | 0.01 | 43.22 | 0 | 0.406 | 0.445 | 1 |
| trauma | ~~ | age |  | 0.009 | 0.007 | 1.431 | 0.152 | -0.003 | 0.022 | 0.022 |
| age | ~~ | age |  | 0.416 | 0.006 | 67.476 | 0 | 0.404 | 0.428 | 1 |
| slope | ~1 |  |  | -0.575 | 0.077 | -7.485 | 0 | -0.728 | -0.423 | -0.575 |
| intercept | ~1 |  |  | 0.778 | 0.079 | 9.858 | 0 | 0.622 | 0.934 | 0.778 |
| intSx | ~1 |  |  | -0.299 | 0.087 | -3.451 | 0.001 | -0.464 | -0.127 | -0.299 |
| trauma | ~1 |  |  | 1.434 | 0.01 | 143.624 | 0 | 1.415 | 1.454 | 2.198 |
| age | ~1 |  |  | 3.003 | 0.01 | 303.619 | 0 | 2.984 | 3.022 | 4.654 |
| Indirect 1 (slope as mediator) | := | a1*b1 | Indirect 1 (slope as mediator) | 0 | 0 | 0.055 | 0.956 | -0.001 | 0.001 | 0 |
| indirect2 (intercept as mediator) | := | a2*b2 | Indirect 1 (intercept as mediator) | 0.005 | 0.002 | 1.965 | 0.049 | 0.001 | 0.01 | 0.003 |
| Total effect | := | c+(a1*b1)+(a2*b2) | total | 0.085 | 0.025 | 3.409 | 0.001 | 0.036 | 0.135 | 0.056 |
| Direct effect | := | c | direct | 0.08 | 0.025 | 3.221 | 0.001 | 0.031 | 0.13 | 0.052 |

Table S11. Male sample: Lavaan output for the multiple mediation model. Notes on model syntax: lhs = left hand side of equation; op = operator; rhs = right hand side of equation; label = mediation path label; ci = 95% confidence interval. IntSx = internalising symptoms. Notes on operators: ~ = regression, ~~ covariance, := specified parameters.

#### Descriptive statistics for male sample

| **Characteristic** | **T1**, N = 4,323*^1^* | **T2**, N = 4,209*^1^* | **T3**, N = 4,233*^1^* | **T4**, N = 4,278*^1^* |
| --- | --- | --- | --- | --- |
| **Age in years** | 9.91 (0.62) | 10.92 (0.64) | 12.01 (0.67) | 12.92 (0.65) |
| **Internalising Symptoms (BPM)** | — | — | — | 1.52 (2.01) |
| *Missing* | — | — | — | 235 |
| **Trauma exposure** |  |  |  |  |
| *0 traumatic events* | 2,806/4,323 (64.9%) | — | — | — |
| *1 traumatic event* | 1,127/ 4,323 (26.1%) | — | — | — |
| *≥2 traumatic events* | 390 / 4,323 (9.0%) | — | — | — |
| **PDS average score** | 1.44 (0.37) | 1.55 (0.43) | 1.82 (0.57) | 2.17 (0.63) |
| **Ethnicity** |  |  |  |  |
| *White* | 2,432 / 4,323 (56.3%) | — | — | — |
| *Black* | 478/4,323 (11.1%) | — | — | — |
| *Hispanic* | 873/4,323 (20.2%) | — | — | — |
| *Asian* | 92 / 4,323 (2.1%) | — | — | — |
| *Other* | 448/4,323 (10.4%) | — | — | — |
| **Parental Education** |  |  |  |  |
| *degree from 4-year college or more* | 2,480/4,316 (57.5%) | — | — | — |
| *some college education* | 1,256/4,316 (29.1%) | — | — | — |
| *HS graduate no college* | 382 / 4,316 (8.9%) | — | — | — |
| *without HS diploma* | 198 / 4,316 (4.6%) | — | — | — |
| *Missing* | 7 | — | — | — |
| **Household income** |  |  |  |  |
| *<$5000* | 106 / 4,010 (2.6%) | — | — | — |
| *$5,000-$11,999* | 121 / 4,010 (3.0%) | — | — | — |
| *$12,000-$15,999* | 81/ 4,010 (2.0%) | — | — | — |
| *$16,000-$24,999* | 175 / 4,010 (4.4%) | — | — | — |
| *$25,000-$34,999* | 215 / 4,010 (5.4%) | — | — | — |
| *$35,000-$49,999* | 338 / 4,010 (8.5%) | — | — | — |
| *$50,000-$74,999* | 563/4,010 (14.0%) | — | — | — |
| *$75,000-$99,999* | 603/4,010 (15.0%) | — | — | — |
| *$100,000-$199,999* | 1,310/4,010 (32.7%) | — | — | — |
| *>$200,000* | 497/4,010 (12.4%) | — | — | — |
| *Missing* | 313 | — | — | — |
| *^1^* Mean (SD); n / N | | | | |

Table S12. Descriptive statistics for male sample. BPM = Brief Problem Monitor. PDS = Pubertal Development Scale, HS = High School.

#### Latent profile analysis results for male sample

| **Class** | **Mean Intercept (SD)** | **Mean Slope (SD)** | **% of sample (N)** |
| --- | --- | --- | --- |
| Early starters | 2.03 (9.24) | 0.12 (2.57) | 4.67 (221) |
| Typical developers | 1.33 (1.59) | 0.37 (0.78) | 51.45 (2437) |
| Slow developers | 1.29 (1.11) | 0.09 (0.57) | 43.89 (2079) |

Table S13. Male sample: Mean intercept and slope values and class sample percentages for a 3-class solution from the latent profile analysis (LPA). Note: Age was centred at the mean age at T1 (9.91 years) in the LPA, and therefore the mean intercept and slope values reported here correspond to when youth are aged 9.91 years. SD = Standard deviation; N participants = 4737; N observations = 17432.

Figure S12. Male sample: A: Pubertal maturation trajectories from baseline (T1) to three-year follow-up (T4), which spans ages 9 to 14 years. Regression lines represent the mean predicted linear growth trajectories for each puberty class with 95% confidence intervals, which are overlaid on individual raw data points (N participants = 4737. N observations = 17432). Classes were derived using latent profile analysis with a random intercept and slope. For plotting purposes, we show the raw data used to derive the latent classes and plot the individual data points overlaid with a mean predicted linear regression for each class. B: Ridge plots showing the variance of average pubertal development scores from T1 to T4 per puberty class. Note: PDS = Pubertal Developmental Scale.

#### Table with latent profile results for male sample

|  | **G** | **conv** | **loglik** | **npm** | **BIC** | **AIC** | **SABIC** | **entropy** | **ICL1** | **ICL2** | **%class1** | **%class2** | **%class3** | **%class4** | **%class5** | **%class6** |
| --- | --- | --- | --- | --- | --- | --- | --- | --- | --- | --- | --- | --- | --- | --- | --- | --- |
| 1 class | 1 | 1 | -12467.49 | 3 | 24960.38 | 24940.99 | 24950.84 | 1 | 24960.38 | 24960.38 | 100 |  |  |  |  |  |
| 2 class | 2 | 1 | -10071.83 | 6 | 20194.43 | 20155.65 | 20175.36 | 0.73 | 21073.32 | 21107.99 | 43.64 | 56.36 |  |  |  |  |
| 3 class | 3 | 1 | -9272.01 | 9 | 18620.19 | 18562.02 | 18591.59 | 0.74 | 19985.73 | 20049.23 | 44.48 | 14.29 | 41.23 |  |  |  |
| 4 class | 4 | 1 | -9029.8 | 12 | 18161.17 | 18083.61 | 18123.03 | 0.7 | 20135.84 | 20264.8 | 26.03 | 32.21 | 36.84 | 4.92 |  |  |
| 5 class | 5 | 1 | -8858.69 | 15 | 17844.33 | 17747.38 | 17796.66 | 0.67 | 20372.13 | 20531.95 | 21.51 | 19.86 | 4.07 | 31.33 | 23.22 |  |
| 6 class | 6 | 1 | -8795.93 | 18 | 17744.19 | 17627.85 | 17686.99 | 0.69 | 20347.9 | 20480.48 | 21.17 | 19.97 | 3.74 | 32.09 | 1.6 | 21.43 |

Table S14. Male sample: Summary fit statistics for classes 1 to 6 for latent profile analysis with no within-class variance. Notes: G = number of classes or profiles in the latent profile analysis; conv = convergence status, where 1 = success and 0 = model did not converge; loglik = log-likelihood of model; npm = number of parameters estimated in the model; BIC = Bayesian Information Criterion; AIC = Akaike Information Criterion; SABIC = Sample-size Adjusted Bayesian Information Criterion; ILC1 = Integrated Complete Likelihood Criterion 1; ILC2 = Integrated Complete Likelihood Criterion 2.

Table S15. Male sample: Latent profile analysis with within-class variance for a three and four class solution, which were the two best fitting models from the latent profile analysis with no within-class variance. Notes: G = number of classes or profiles in the latent profile analysis; conv = convergence status, where 1 = success and 0 = model did not converge; loglik = log-likelihood of model; npm = number of parameters estimated in the model; BIC = Bayesian Information Criterion; AIC = Akaike Information Criterion; SABIC = Sample-size Adjusted Bayesian Information Criterion; ILC1 = Integrated Complete Likelihood Criterion 1; ILC2 = Integrated Complete Likelihood Criterion 2.

|  | G | conv | loglik | npm | BIC | AIC | SABIC | entropy | ICL1 | ICL2 | %class1 | %class2 | %class3 | %class4 |
| --- | --- | --- | --- | --- | --- | --- | --- | --- | --- | --- | --- | --- | --- | --- |
| 3 class | 3 | 1 | -8740.37 | 14 | 17599.22 | 17508.73 | 17554.73 | 0.64 | 19455.38 | 19348.37 | 4.67 | 43.89 | 51.45 |  |
| 4 class | 4 | 1 | -8715.57 | 18 | 17583.47 | 17467.14 | 17526.27 | 0.61 | 20113.32 | 20162.85 | 48.09 | 4.39 | 8.57 | 38.95 |

#### Latent profile analysis fit statistics with within-class variance for male sample

#### References

MacSweeney, N., Allardyce, J., Edmondson-Stait, A., Shen, X., Casey, H., Chan, S. W. Y., Cullen, B., Reynolds, R. M., Frangou, S., Kwong, A. S. F., Lawrie, S. M., Romaniuk, L., & Whalley, H. C. (2023). The role of brain structure in the association between pubertal timing and depression risk in an early adolescent sample (the ABCD Study®): A registered report. *Developmental Cognitive Neuroscience*, *60*, 101223. https://doi.org/10.1016/j.dcn.2023.101223

Mendle, J., Beltz, A. M., Carter, R., & Dorn, L. D. (2019). Understanding Puberty and Its Measurement: Ideas for Research in a New Generation. *Journal of Research on Adolescence*, *29*(1), 82–95. https://doi.org/10.1111/jora.12371

Petersen, A. C., Crockett, L., Richards, M., & Boxer, A. (1988). A self-report measure of pubertal status: Reliability, validity, and initial norms. *Journal of Youth and Adolescence*, *17*(2), 117–133. https://doi.org/10.1007/BF01537962

Vijayakumar, N., Whittle, S., & Silk, T. J. (2023). Corticolimbic connectivity mediates the relationship between pubertal timing and mental health problems. *Psychological Medicine*, 1–11. https://doi.org/10.1017/S0033291723001472
